# Roseobacters in a Sea of Poly- and Paraphyly: Whole Genome-Based Taxonomy of the Family *Rhodobacteraceae* and the Proposal for the Split of the “Roseobacter Clade” Into a Novel Family, *Roseobacteraceae* fam. nov.

**DOI:** 10.3389/fmicb.2021.683109

**Published:** 2021-06-25

**Authors:** Kevin Y. H. Liang, Fabini D. Orata, Yann F. Boucher, Rebecca J. Case

**Affiliations:** ^1^Department of Biological Sciences, University of Alberta, Edmonton, AB, Canada; ^2^Singapore Centre for Environmental Life Sciences Engineering, Nanyang Technological University, Singapore, Singapore; ^3^Saw Swee Hock School of Public Health, National University Singapore, Singapore, Singapore; ^4^School of Biological Sciences, Nanyang Technological University, Singapore, Singapore

**Keywords:** *Rhodobacteraceae*, *Roseobacteraceae* fam. nov., roseobacter clade, genomic taxonomy, whole-genome phylogeny, digital DNA–DNA hybridization, average nucleotide identity, average amino acid identity

## Abstract

The family *Rhodobacteraceae* consists of alphaproteobacteria that are metabolically, phenotypically, and ecologically diverse. It includes the roseobacter clade, an informal designation, representing one of the most abundant groups of marine bacteria. The rapid pace of discovery of novel roseobacters in the last three decades meant that the best practice for taxonomic classification, a polyphasic approach utilizing phenotypic, genotypic, and phylogenetic characteristics, was not always followed. Early efforts for classification relied heavily on 16S rRNA gene sequence similarity and resulted in numerous taxonomic inconsistencies, with several poly- and paraphyletic genera within this family. Next-generation sequencing technologies have allowed whole-genome sequences to be obtained for most type strains, making a revision of their taxonomy possible. In this study, we performed whole-genome phylogenetic and genotypic analyses combined with a meta-analysis of phenotypic data to review taxonomic classifications of 331 type strains (under 119 genera) within the *Rhodobacteraceae* family. Representatives of the roseobacter clade not only have different environmental adaptions from other *Rhodobacteraceae* isolates but were also found to be distinct based on genomic, phylogenetic, and *in silico*-predicted phenotypic data. As such, we propose to move this group of bacteria into a new family, *Roseobacteraceae* fam. nov. In total, reclassifications resulted to 327 species and 128 genera, suggesting that misidentification is more problematic at the genus than species level. By resolving taxonomic inconsistencies of type strains within this family, we have established a set of coherent criteria based on whole-genome-based analyses that will help guide future taxonomic efforts and prevent the propagation of errors.

## Introduction

Taxonomy is the science of characterizing, naming, and classifying organisms based on shared traits meaningful to their ecology, physiology, and evolutionary history (Wayne et al., 1987). Microbial taxonomy has changed substantially in the past few decades along with the development of new technologies and has embraced a polyphasic approach (i.e., phenotypic, genotypic, and phylogenetic) (Vandamme et al., 1996). This has led to our evolved understanding of bacterial diversity. Many earlier taxonomic classifications have been re-evaluated and modified (Parks et al., 2018, 2020), as they are hypotheses that should be continuously verified when better techniques become available (Garrity, 2016).

Examination of phenotypic traits is the oldest tool for bacterial characterization and classification (Tindall et al., 2010). Even with the availability of many standardized high-throughput tests (Shea et al., 2012; Sneath, 2015), bacteria are rarely classified based on common phenotypes. This is partly because bacteria are metabolically and phenotypically diverse and at times atypical phenotypic tests are required for species with specialized adaptive traits (Tindall et al., 2010). Also, phenotypes can vary even between isolates of the same species. Despite these limitations, phenotypic testing is still necessary as it highlights important ecological roles and/or clinical traits. In this regard, next-generation sequencing has significantly advanced phenotypic predictions. It is now possible to efficiently analyze a large number of distantly related isolates to identify potentially distinguishing phenotypic traits from their genome sequences (Amaral et al., 2014; Kanehisa et al., 2016). Not only do *in silico*-predicted phenotypes closely resemble observed phenotypic traits (Aziz et al., 2008; Kanehisa et al., 2016), these predictions have also been shown to match closely with known ecological adaptations (Simon et al., 2017). Additionally, in examining broader taxonomic levels that deal with a large number of diverse and distantly related isolates, it is increasingly more difficult to identify universally present phenotypic traits. It is not uncommon for taxonomic descriptions to include traits that are only shared by some representatives (Wells et al., 1987; Garrity et al., 2015a; Orata et al., 2016). Therefore, it is more important to identify unifying traits that are likely ancestral and reflect a common evolutionary history (Philippot et al., 2010; Simon et al., 2017). Results from *in silico* data can then guide subsequent laboratory experiments, which will significantly reduce the time and cost of phenotypic characterizations as only a handful of phenotypic traits are required to be verified (Orata et al., 2016).

Genotypic and phylogenetic analyses are the other two important pillars of polyphasic taxonomy (Wayne et al., 1987). The earlier methods include G+C content deviation, 16S rRNA gene analyses, and DNA–DNA hybridization (DDH). DDH is the proposed gold standard for species delineation. It is widely accepted that isolates exhibiting ≥70% DDH belong to the same species (Wayne et al., 1987). However, DDH is time and labor intensive, notoriously difficult to reproduce, and carried out reliably by a few laboratories only (Gevers et al., 2005). As such, DDH was eventually replaced by 16S rRNA gene sequence analysis (Tindall et al., 2010). Although this allows for a rapid characterization of prokaryotes based on a universally distributed gene marker, 16S rRNA gene sequences often lack resolution when compared with DDH. High 16S rRNA gene similarity values (i.e., 97–99%) do not necessarily guarantee high DDH (Wang X. et al., 2014), highlighting the discrepancy between the two methods. It is recommended that 16S rRNA gene analysis should only serve as a preliminary guide to whether more in-depth genomic and phylogenetic analyses are required (Tindall et al., 2010). However, 16S rRNA gene-based phylogeny alone is still commonly used to fulfill the phylogenetic aspect of polyphasic taxonomy (Kim et al., 2010; Baek et al., 2015; Shin et al., 2017).

Advancements in next-generation whole-genome sequencing has provided the basis to develop more accurate genetic and phylogenetic methods and has provided us with tangible standards for systematic classifications, more so for the species level than higher ranks. One such method is average nucleotide identity (ANI) between genomes, which is primarily used for species-level delineation of taxa. It was determined that 95% ANI corresponds to 70% DDH and is proposed as the species cutoff (Goris et al., 2007). Additionally, DDH can now be calculated *in silico* (digital DDH or dDDH) while retaining the 70% species cutoff as in traditional DDH (Meier-Kolthoff et al., 2013). As ANI and dDDH are reproducible and easily scaled to analyze hundreds of isolates, it is becoming standard practice for species delineation (Orata et al., 2016, 2018; Dees et al., 2017; Wirth and Whitman, 2018). For higher taxonomic ranks, a proposed method for classification is average amino acid identity (AAI), which is similar to ANI but considers amino acid sequences instead. AAI is more suitable than ANI to assess higher ranks among more distantly related species because amino acids do not reach mutational saturation as quickly as nucleotides (Qin et al., 2014).

Various genomic metrics were employed in this study for detailed phylogenetic and genomic analyses, supplemented with phenotypic data, to identify and fix potential misclassifications within the *Rhodobacteraceae* family (order *Rhodobacterales*). This family is metabolically, phenotypically, and genotypically diverse (Garrity et al., 2015b). *Rhodobacteraceae* was circumscribed based on 16S rRNA gene analysis and was named after the first described genus, *Rhodobacter* (Garrity et al., 2015b). A part of *Rhodobacteraceae* is the roseobacter clade, historically known as the marine *Agrobacterium* (Uchino et al., 1998) and belong to one of the most readily cultivated groups of marine bacteria (Buchan et al., 2005). The roseobacters can consist of up to 20% of coastal marine bacterial populations, making it one of the most abundant groups of marine bacteria (Moran et al., 2007). In addition, it contains isolates capable of both pathways for dimethylsulfoniopropionate (DMSP) degradation – DMSP demethylation and DMSP cleavage (Luo and Moran, 2014). These pathways utilize DMSP in different ways and both play crucial ecological and environmental roles (Todd et al., 2007; Reisch et al., 2011; Moran et al., 2012). Taxonomic classifications within *Rhodobacteraceae* continue to rely heavily on 16S rRNA gene phylogeny and misclassifications have been a reoccurring problem, underscoring the instability of classifications based on the 16S rRNA gene. In this study, the abundance of high-quality whole-genome sequencing data was used to perform large-scale phylogenomic analyses on type strains to resolve taxonomic inconsistencies. This establishes a set of taxonomically correct reference material that can help guide future taxonomic efforts and prevent the propagation of error. In addition, type strains also provide phenotypic data for a meta-analysis allowing us to follow the polyphasic approach more closely.

## Materials and Methods

### Genome Dataset Used in This Study

Whole-genome sequences from 342 type strains within *Rhodobacteraceae* were obtained from the National Center for Biotechnology Information GenBank database on January 13th, 2019 (Supplementary Table 1). In addition, sequences from three strains of *Agrobacterium tumefaciens* (order *Rhizobiales*, class *Alphaproteobacteria*; accession numbers CP011247.1, APLP01, and APJV01) were used as outgroup for all phylogenetic analyses. Plasmid sequences were excluded from analyses where possible. All accessions numbers for the genomes used are listed in Supplementary Tables 1, 2.

### Genome Annotation and Core Genome Identification

An important consideration when reconstructing a core-genome phylogeny is the quality of the assembled genomes used. Poor sequence quality or assembly will affect gene annotations and the number of core genes identified (Moura et al., 2017), which will ultimately affect the reconstruction of the phylogeny. We addressed this issue by ensuring our genome sequences are complete or nearly complete (i.e., ≥95% complete) with low levels of contamination (≤5%) as outlined in CheckM (Parks et al., 2015), which assess these criteria based on the presence and the number of copies of a set of well-defined core genes. As a result, we excluded 11 genomes from our initial dataset leaving us with 331 genomes (Supplementary Table 3).

All 331 high-quality *Rhodobacteraceae* genomes, plus the three *A. tumefaciens* genomes, were annotated using RAST 2.0 (Aziz et al., 2008) or Prodigal 2.6.3 (Hyatt et al., 2010). Core genes, which are genes present in all genomes of interest, were identified using the Bacterial Pan Genome Analysis pipeline (Chaudhari et al., 2016), which employs the USearch gene clustering algorithm (Edgar, 2010). There were 140 core genes identified from the 331 genomes.

### 16S rRNA Gene and Core-Genome Phylogenetic Analyses

A single copy of the full-length 16S rRNA gene was extracted from all the genomes. These sequences were then aligned with MUSCLE 3.8.31 using default parameters (Edgar, 2004). The final alignment (with 1,628 nucleotide positions) was used to reconstruct a maximum-likelihood phylogenetic tree using RAxML 8.2.11 (Stamatakis, 2014). The GTR (general time reversible) nucleotide substitution model and gamma model of rate heterogeneity were used. Robustness of branching was estimated with 1,000 bootstrap replicates. Applying the majority rule for consensus trees, where a branching pattern that occurs at least 50% of the time is adopted (Russo and Selvatti, 2018), clades with less than 50% bootstrap support were collapsed to polytomies using iTOL 5.7 (Letunic and Bork, 2019).

For every set of core genes, the amino acid sequences were aligned with MUSCLE 3.8.31 using default parameters (Edgar, 2004). The core gene alignments were then concatenated using Geneious 8.1.8 (Kearse et al., 2012). The final alignment (with 71,480 amino acid positions) was used to reconstruct a maximum-likelihood core-genome phylogenetic tree using RAxML 8.2.11 (Stamatakis, 2014) with the PROTGAMMAAUTO option for automatic model selection. Robustness of branching was estimated with 100 bootstrap replicates, and clades with less than 50% bootstrap support were collapsed, as described above.

### Species-Level Delineation

Phylogenetically, the minimum requirement for a set of isolates to be considered as part of the same species is that they must form a monophyletic clade (Rosselló-Móra and Amann, 2001), for which we assessed using a core-genome phylogeny. Here, dDDH and ANI were also used for species delineations; dDDH was calculated with the online Genome-to-Genome Distance Calculator (Meier-Kolthoff et al., 2013) and ANI was calculated using JSpecies (Richter and Rosselló-Móra, 2009), using default parameters for both.

To identify any species-level misclassification, dDDH was calculated for isolates belonging to the same genus. For polyphyletic genera, only isolates within the same monophyletic clade were compared as it is highly unlikely for isolates to share more than 70% dDDH values if they are not monophyletic in a tree. For any genus where species-level misclassifications were identified based on dDDH, ANI was also calculated for those comparisons. Isolates that met or surpassed the species thresholds for both dDDH (70%) and ANI (95%) that were also monophyletic in the core-genome tree were considered to belong to the same species.

### Assessing Genomic Similarities for the Genus and Family Levels

Amino acid identity and codon position (CP) similarities were used to assess genus- and family-level genomic similarities. CPs can be used to assess genus- or even higher-level classifications due to slower mutation rates as they are part of the coding sequence relating to amino acids. We therefore calculated CP similarities of all the core genes identified as a separate metric. In addition, evolutionary distance based on the core-genome phylogenetic tree was quantified using patristic distance (PD), which is the sum of branch lengths between two nodes of a phylogenetic tree and is used to evaluate, among other things, evolutionary rate and genetic distances (Fourment and Gibbs, 2006). AAI was calculated using CompareM (Parks, 2014). CP similarities and PD were calculated using translatorX (Abascal et al., 2010) and Geneious 8.1.8 (Kearse et al., 2012), respectively.

### Genus-Level Delineation Based on Genomic, Phenotypic, and Phylogenetic Data

Mono-, para-, and polyphyletic genera were identified based on the core-genome phylogenetic tree. To assess if any currently recognized genera are misclassified, AAI, CP similarities, and PD were calculated by comparing all strains within and between monophyletic genera, excluding strains in any para- and polyphyletic genera. The significance of each genomic metric was assessed by applying the Mann–Whitney *U* test (McKnight and Najab, 2010).

For paraphyletic genera, genomic metrics were calculated for within and between genera comparisons within these clades. These values were compared to those obtained from within and between recognized monophyletic genera comparisons (Supplementary Table 4) to determine whether genomic similarities among those being merged fell within the expected range of within-genus comparisons. If so, the first described genus within the clade, referred to as the primary genus, retained the genus designation as per rule 38 of the *International Code of Nomenclature of Prokaryotes: Prokaryotic Code* (Parker et al., 2019), and other genera within that clade were combined with the primary genus. On the other hand, if values fell outside the expected range, the monophyletic cluster containing the type species of the primary genus retained its genus designation and all other clades were reclassified as necessary in accordance with the monophyletic rule of taxonomic classification.

For polyphyletic genera, the clade containing the type species of the genus, referred to as the primary clade, retained the genus designation as per rule 39a of the *Prokaryotic Code* (Parker et al., 2019). Whether the other clades were given novel genera designations or combined with existing genera was determined based on phenotypic, genomic, and phylogenetic data. Phenotypic information was collected from the *Bergey’s Manual* or the original isolation papers.

### Family-Level Delineation Based on Genomic, Phenotypic, and Phylogenetic Data

Genomic similarities at the family level were assessed based on AAI, CP similarities, and PD, as described above. Core-genome phylogeny was used to assess phylogenetic relationships. Environment of isolation and salinity level were collected from original isolation papers. Phenotypic traits characteristic of the roseobacter clade and marker genes used to assess presence/absence of these traits were identified from current literature. Using annotated genomes, the presence/absence of major pathways were assessed for the two major clades found within our core genome tree. Significance of the differences in proportion of these pathways between the two clades was assessed using proportion *Z*-test.

## Results and Discussion

### The 16S rRNA Gene Phylogeny Provides Little Resolution of *Rhodobacteraceae* Relative to the Core-Genome Phylogeny

**FIGURE 1:**
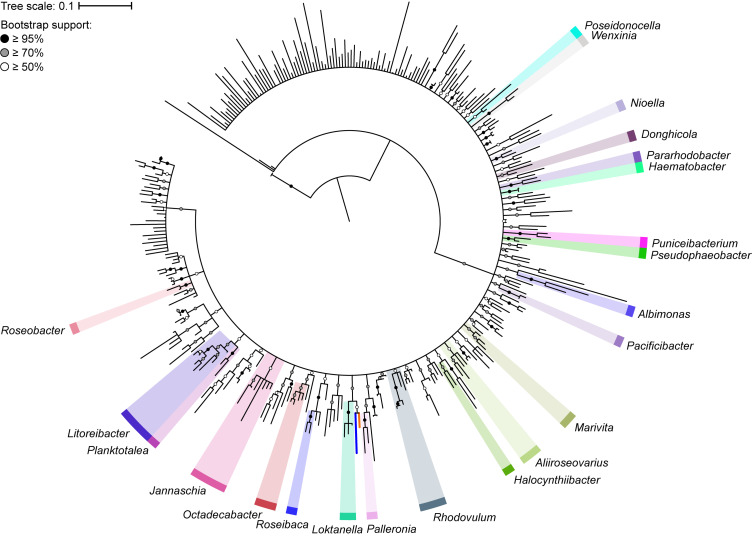
Phylogenetic tree of 331 *Rhodobacteraceae* type strains based on the full-length 16S rRNA gene (1,698 nucleotide positions). The maximum-likelihood tree was reconstructed using RAxML 8.2.11 with the GTRGAMMA model and rooted with three *A. tumefaciens* strains. Branch support is evaluated with 1,000 bootstrap replicates and indicated on the nodes as black (≥95%), gray (≥70%), or white (≥50%) circles; nodes with <50% bootstrap support are collapsed. The scale bar represents nucleotide substitutions per site. All monophyletic genera based on this collapsed 16S rRNA gene tree are highlighted. The blue branch marks the position of *Y. vestfoldensis*; the orange branch marks the position of *F. marinus*.

The 16S rRNA gene has played a major role in the taxonomic classification of *Rhodobacteraceae* (Garrity et al., 2015b). The largest lineage within *Rhodobacteraceae*, the marine roseobacter clade, is defined by having representatives that share >89% 16S rRNA gene sequence identity (Buchan et al., 2005). To determine the impact of using the 16S rRNA gene as the main molecular marker for naming new species and genera within this family, we reconstructed the phylogenetic tree of 331 type strains using full-length 16S rRNA gene sequences, which are recommended for use in phylogenetic and taxonomic studies (Tindall et al., 2010). As expected, the 16S rRNA gene-based tree has poor resolution and low bootstrap support overall (Figure 1). This is more evident when clades with less than 50% bootstrap support were collapsed, resulting in a poorly resolved tree backbone. The inadequacy of the 16S rRNA gene for use in genus-level classification is highlighted by the fact that only 22 of 119 genera in the entire family are monophyletic with strong bootstrap support.

**FIGURE 2:**
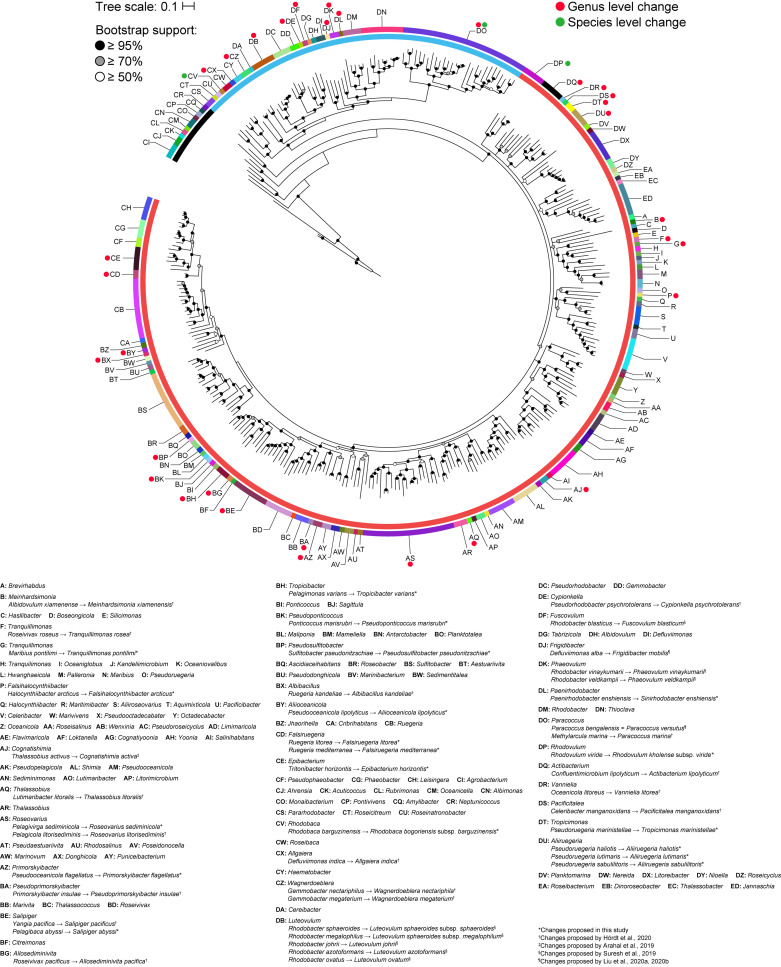
Core-genome phylogenetic tree of 331 *Rhodobacteraceae* type strains based on the concatenated alignment of 140 core protein-coding genes (71,480 amino acid positions). The maximum-likelihood tree was reconstructed using RAxML 8.2.11 with the PROTGAMMAAUTO option for automatic model selection and rooted with three *A. tumefaciens* strains. Branch support is evaluated with 100 bootstrap replicates and indicated on the nodes as black (≥95%), gray (≥70%), or white (≥50%) circles; nodes with <50% bootstrap support are collapsed. The scale bar represents amino acid substitutions per site. The inner ring represents the two major lineages within the family and the outer ring represents monophyletic clades. Red and green dots represent genus and species level changes, respectively, from this study or previous works (Arahal et al., 2019; Suresh et al., 2019; Hördt et al., 2020; Liu et al., 2020a, b).

A core genome approach was employed to reconstruct and resolve phylogenetic relationships. In a previous study, a core-genome phylogeny of the roseobacter clade was reconstructed using 108 core housekeeping genes (Luo and Moran, 2014). To determine the phylogenetic affiliation of this clade within the family *Rhodobacteraceae*, the phylogeny of the entire family was reconstructed in a subsequent study using 208 core genes from 106 strains (Simon et al., 2017). In this study, the core-genome phylogeny of the family was reconstructed using 140 core genes from a dataset of more than three times as large, providing a more complete picture of the phylogenetic framework of the *Rhodobacteraceae* family. As expected, phylogenetic relationships are much better resolved in the core-genome phylogeny than with the 16S rRNA gene alone, with a well-defined backbone (Figure 2). Based on this core-genome tree, *Rhodobacteraceae* can be divided into two major monophyletic lineages, one of which consists of the roseobacter clade and is composed primarily of organisms found in marine environments (Buchan et al., 2005) (Supplementary Table 2). It should be noted that the 16S rRNA gene phylogeny was unable to resolve these two major lineages (Figure 1), meaning it would be difficult to even determine which lineage an isolate belongs let alone its genus or species affiliation using this gene. In addition, genera that were not monophyletic in the 16S rRNA gene tree (e.g., *Yoonia*, *Leisingera*, and *Phaeobacter*) are monophyletic in the core-genome tree with strong bootstrap support, consistent with prior studies (Wirth and Whitman, 2018) (Figure 2). Based on our phylogenetic analyses, we identified several polyphyletic (*Albidovulum, Celeribacter, Defluviimonas, Gemmobacter, Lutimaribacter, Maribius, Oceanicola, Ponticoccus, Primorskyibacter, Pseudooceanicola, Pseudorhodobacter, Pseudoruegeria, Rhodobacter, Roseivivax, Ruegeria, Sulfitobacter, Thalassobius*) and paraphyletic (*Actibacterium, Epibacterium, Paracoccus, Roseovarius, Salipiger, Tropicibacter, Tropicimonas*) genera.

### Evaluation of Species Designation Within Monophyletic Genera

Unlike higher taxonomic ranks, there are clear genomic and phylogenetic criteria for species-level delineation. dDDH and ANI are two common genomic metrics which use 70% and 95% as the species threshold, respectively (Richter and Rosselló-Móra, 2009; Meier-Kolthoff et al., 2013). Phylogenetically, all isolates belonging to the same species must also be monophyletic (Rosselló-Móra and Amann, 2015). We propose species-level taxonomic changes only for cases where dDDH, ANI, and phylogenetic data support the merging of two species.

From all comparisons, only three pairs of species had more than 70% dDDH and 95% ANI values. These were between (1) *Paracoccus bengalensis* and *Paracoccus versutus*, (2) *Luteovulum sphaeroides* and *Luteovulum sphaeroides* subsp. *megalophilum*, and (3) *Rhodovulum kholense* and *Rhodovulum viride*. The conflict between the pairs of *Paracoccus* or *Luteovulum* has already been resolved previously (Figure 2), and no further reclassifications are warranted. *P. bengalensis* is now a later heterotypic synonym of *P. versutus* (Liu et al., 2020b). *Luteovulum sphaeroides* subsp. *megalophilum* was formerly known as *Rhodobacter megalophilus* and reclassified as a subspecies of *Luteovulum sphaeroides* (formerly, *Rhodobacter sphaeroides*) (Suresh et al., 2019).

On the other hand, there is still a need to address the conflict between *R. kholense* and *R. viride*. In addition to dDDH and ANI values surpassing species-level cutoffs (Supplementary Figure 1), both species share several phenotypic traits. They can utilize glucose, glutamate, malate, and mannitol as carbon sources; have the ability for dark aerobic growth; and have similar G+C content (67.8% and 67.6%, respectively). They differ with each other phenotypically in terms of NaCl and pH growth ranges; some vitamin requirements; as well as the utilization of carbon sources such as propionate, valerate, and fumarate, among others (Supplementary Tables 2, 5) (Srinivas et al., 2014). We therefore propose *R. viride* as a subspecies of *R. kholense* and be reclassified accordingly as *Rhodovulum kholense* subsp. *viride* subsp. nov.

It is worthwhile to note that despite minor differences between the dDDH and ANI values calculated in this study and those from previous studies (Suresh et al., 2019; Liu et al., 2020b), the same conclusions can be made. This underscores the reliability and replicability of using these genome-based metrics for species-level delineations.

### Genome-Guided Genus-Level Reclassifications Supported by Phylogenetic Data

Taxonomic classifications at the genus and higher levels are more difficult, as standardized metrics or guidelines are lacking. Although attempts were made to establish genomic standards for genus-level classification, there has yet to be a consensus on analyses and applicable cutoff values (Luo et al., 2014; Orata et al., 2018; Wirth and Whitman, 2018). As a result, a polyphasic approach that includes phenotypic and phylogenetic data is favored for assigning taxa above the species level. However, it should be noted that although genome-based similarity analyses alone are not sufficient to justify genus-level reclassifications, the relative ease in analyzing hundreds of isolates using a variety of metrics makes these methods effective initial approaches for identifying potential misclassifications in large datasets. These can be further examined from a phylogenetic and phenotypic perspective; both of which are more time-consuming and computationally intensive.

In the past, genus definition relied heavily on 16S rRNA gene sequence analyses (Ludwig et al., 1993; Labrenz et al., 2000; Wang D. et al., 2014). As such, genomically dissimilar organisms are sometimes grouped into the same genus because distantly related organisms may still have similar 16S rRNA gene sequences. For example, *Yoonia vestfoldensis* and *Flavimaricola marinus* (Wirth and Whitman, 2018) both share a 96% 16S rRNA gene identity and are monophyletic based on the 16S rRNA gene phylogenetic tree with more than 50% bootstrap support (Figure 1 and Supplementary Table 4). However, relative to other *Yoonia* species, *Y. vestfoldensis* exhibits a lower AAI, as well as 1st, 2nd, and 3rd CP similarity values and higher PD when compared to *F. marinus* (Supplementary Table 4). If genus classifications were assigned based solely on 16S rRNA gene analyses, these two isolates would be grouped into the same genus despite being genomically dissimilar. This was indeed the case in the past (Van Trappen et al., 2004; Jung et al., 2016); however, genomic similarity analyses showed these isolates do not belong to the same genus and further phylogenetic and phenotypic analyses corroborated these results, resulting into their split (Wirth and Whitman, 2018). This highlights the importance of genomic similarity analyses as efficient methods for quickly identifying potential misclassifications that can help guide subsequent analyses.

**FIGURE 3:**
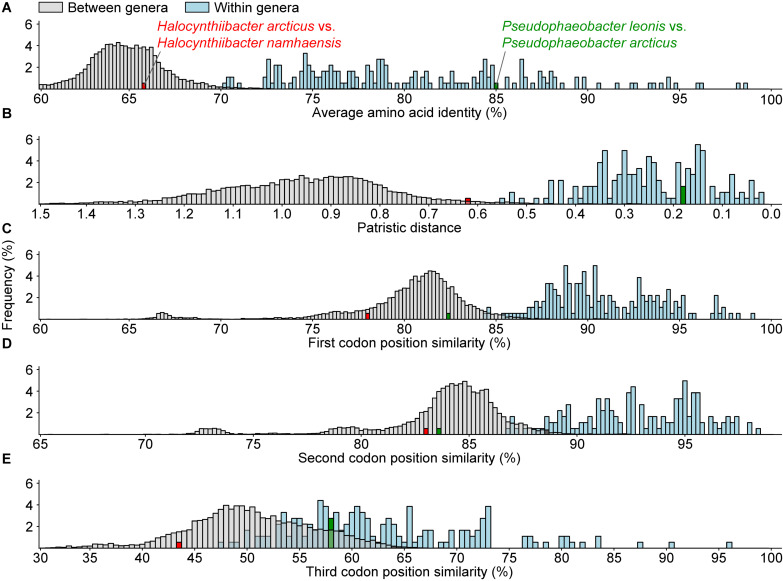
Histogram of **(A)** average amino acid, **(B)** patristic distance, and **(C–E)** 1st, 2nd, and 3rd codon position similarities for all between (gray) or within (blue) recognized monophyletic genera. The distributions for within and between genera comparisons for all metrics are statistically significant (*p* < 0.05) based on the Mann–Whitney *U* test. The red and the green bars represent two atypical within genus comparisons.

Genomic and phylogenetic metrics – AAI, CP similarity, PD – were therefore used to determine if there are any misclassifications among currently recognized monophyletic genera within the two dominant lineages (Figure 2 and Supplementary Tables 4, 6). In general, species within the same genus are more similar to each other than species between genera, as values for within genera comparisons are statistically different from between genera comparisons (Figure 3 and Supplementary Table 4). It is also worth noting that between and within genera comparisons always have some overlaps for all metrics considered. These overlaps are expected, as even closely related genera can have different evolutionary rates due to differences in response to evolutionary and ecological processes (Ramette and Tiedje, 2007). This means genera will contain species of varying degrees of diversity. This overlap is the primary reason why establishing a single universal genus level boundary is difficult if not impossible.

All within-genera comparisons have AAI values above 70% with only one exception – the comparison between *Halocynthiibacter arcticus* (Baek et al., 2015) and *Halocynthiibacter namhaensis* (Kim et al., 2014) at 65.8% (Figure 3A), the only two named species within this genus. Other genomic metrics, PD (Figure 3B) and CP similarities (Figures 3C–E) also show a similar pattern where these two isolates have values consistent with those observed for between genera comparisons rather than within genus comparisons. Together with the core-genome phylogeny (Figure 2), these metrics collectively show that *H. arcticus* and *H. namhaensis* are genomically and phylogenetically distinct and should in fact be considered as parts of different genera.

It is likely that *H. articus* was misclassified, as it was originally circumscribed to *Halocynthiibacter* based solely on 16S rRNA gene sequence analyses (Baek et al., 2015). Consistent with Baek et al. (2015), *H. articus* does have the highest 16S rRNA identity with *H. namhaensis* at 96.6% (Supplementary Table 4); however, *H. articus* shares a similarly high level of 16S rRNA identity with *Pseudopelagicola gijangensis* at 96.1%. In addition, within the dataset used in this study, the ranges of 16S rRNA sequence identity for within and between genera comparisons are 93.3–99.9% and 84.1–97.9%, respectively. 16S rRNA identity of 96.6% is therefore not sufficient to support the placement of *H. arcticus* with *H. namhaensis* in the same genus. The separation of these two species into different genera is also supported by differences in phenotypic traits previously identified (e.g., difference in temperature growth range, salt tolerance, pH tolerance, enzymatic activities, and carbon metabolism) (Supplementary Table 5) (Baek et al., 2015). As such, we propose to move *H. arcticus* to a new genus, *Falsihalocynthiibacter* gen. nov., with *Falsihalocynthiibacter arcticus* comb. nov. as the type species.

It is worth mentioning that although based on 1st and 2nd CP similarities alone, *Pseudophaeobacter leonis* and *Pseudophaeobacter arcticus* also seem to belong to different genera (Figures 3C,D), but unlike the *Halocynthiibacter* species, AAI, PD, and the 3rd CP similarity for these two *Pseudophaeobacter* species are within the expected range (Figures 3A,B,E). As genomic metrics are providing conflicting results for these two isolates, a definitive decision cannot be made until additional in-depth genomic, phylogenetic, and phenotypic characterization is done for both species, ideally when more isolates become available.

### Reclassifications at the Genus Level: Addressing Paraphyletic Genera

**FIGURE 4:**
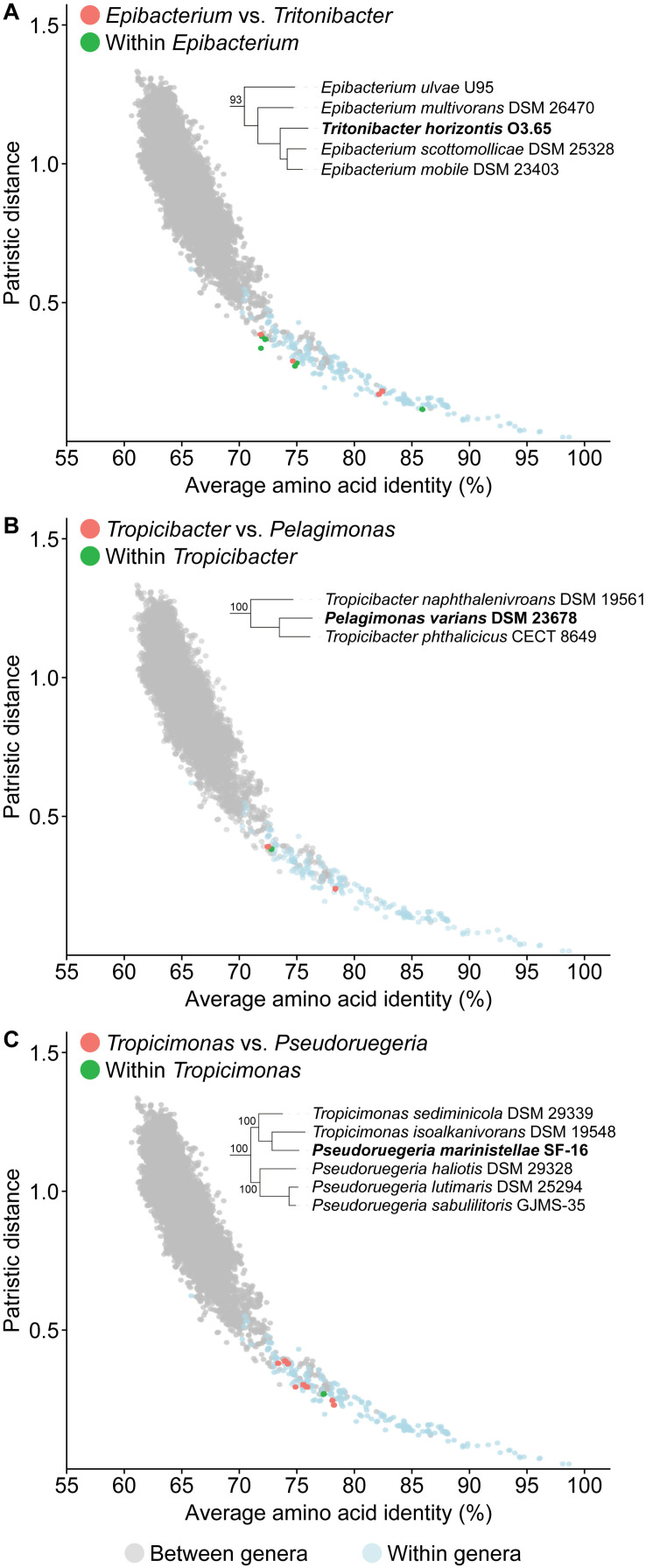
Dot plot for patristic distance (PD) against average amino acid identity (AAI) highlighting the comparisons of representative paraphyletic genera **(A)** *Epibacterium*, **(B)** *Tropicibacter*, **(C)** *Tropicimonas*, and closely related organisms. PD and AAI comparisons for between (orange) or within (green) genera of interest are shown. PD and AAI comparisons for all between (gray) or within (blue) recognized monophyletic genera are included as reference. The corresponding phylogenetic trees are subsets of the core-genome tree (Figure 2). Names in bold are organisms causing paraphyly of genera of interest.

One of the goals of this study is to resolve all non-monophyletic genera in this family by using genomic analysis to guide polyphasic taxonomy. A total of seven paraphyletic genera were identified based on our core-genome phylogeny, as they form monophyletic clades with one or more species of a different genus (16 genera involved in total) with strong bootstrap support (Figure 2 and Supplementary Table 7). Ultimately, only seven genera should retain their designation as all conflicting genera within a clade should be merged to their corresponding primary genus (i.e., the first described genus of that clade). For each clade, PD and AAI comparisons are all within the range observed for typical within genus comparisons (Figure 4, Supplementary Figure 2, and Supplementary Table 8), providing genomic support for the merging of these genera.

An independent study published recently based on the Genome BLAST Distance Phylogeny (GBDP), 16S rRNA gene analyses, and phenotypic data has proposed some genus-level reclassifications to address some of the paraphyletic genera also identified in our study (Hördt et al., 2020). These include the transfer of *Pelagicola litorisediminis* to the genus *Roseovarius* (Supplementary Figure 2A), *Yangia pacifica* to the genus *Salipiger* (Supplementary Figure 2B), *Confluentimicrobium lipolyticum* to the genus *Actibacterium* (Supplementary Figure 2C), and *Methylarcula marina* to the genus *Paracoccus* (Supplementary Figure 2D). However, several paraphyletic genera remained, and two genus-level reclassifications proposed from that study conflicted with our analyses.

The first conflict is regarding the placement of *Tritonibacter*. Hördt et al. (2020) proposed to move *Epibacterium* except the type species (*Epibacterium ulvae*) to the genus *Tritonibacter*. This is largely because *E. ulvae* forms a monophyletic clade with *Tritonibacter* with only 73% bootstrap support while the remaining representatives of the *Epibacterium* genus form a monophyletic clade with *Tritonibacter* with 100% bootstrap support (Hördt et al., 2020). Our core-genome tree shows the same phylogenetic relationships (Figure 2); however, *E. ulvae* forms a monophyletic clade with *Epibacterium* and *Tritonibacter* with 93% bootstrap support (Figure 4A). Although GBDP is a rapid method to reconstruct phylogenies, it is not as exhaustive as the maximum-likelihood approach used here and is more susceptible to artefacts caused by changes in evolutionary rates and G+C content (Yokono et al., 2018; Zielezinski et al., 2019). Taking this phylogeny together with our genomic analyses (Supplementary Table 8) and phenotypic data (Supplementary Table 5), we recommend the transfer of *Tritonibacter horizontis* to *Epibacterium*. This is the more parsimonious solution requiring only one species name change, as opposed to the previous proposal which requires four species name changes (Hördt et al., 2020).

The second conflict between our study and that of Hördt et al. (2020) is regarding the placement of *Pelagimonas varians*. Based on their GBDP tree, *Tropicibacter* appears as a polyphyletic genus, where *Tropicibacter phthalicicus* forms a monophyletic clade with *P. varians*, meanwhile *Tropicibacter naphthalenivorans* forms a different monophyletic clade with six other genera (Hördt et al., 2020). However, our phylogenetic analysis shows *P. varians* forming a monophyletic clade with *T. phthalicicus* and *T. naphthalenivorans* with 100% bootstrap support making *Tropicibacter* a paraphyletic genus (Figure 4B). Our placement is more consistent with other studies where it was also shown that both species are monophyletic (Iwaki et al., 2012; Lucena et al., 2013). This suggests that the most logical approach is to resolve the paraphyletic genus by transferring *P. varians* to the genus *Tropicibacter*. This change is also supported by our genomic analyses (Supplementary Table 8) and phenotypic data (Supplementary Table 5).

Unlike other paraphyletic genera we have identified, *Tropicimonas* is monophyletic with four *Pseudoruegeria* strains (Figure 4C), which are part of a polyphyletic genus (the implications of this polyphyly are discussed further below). Genomic similarities between the two *Tropicimonas* species and the four *Pseudoruegeria* species are within the range expected for within-genus comparisons. Phylogenetically, it would resolve this paraphyletic genus to move the two *Tropicimonas* species into the genus *Pseudoruegeria*, as the latter was described before *Tropicimonas*. This, however, is not the most parsimonious solution as it results in two name changes. Instead, it is proposed that *Pseudoruegeria marinistellae* be moved into the genus *Tropicimonas* and be renamed accordingly as *Tropicimonas marinistellae* comb. nov., as supported by phylogenetic, genomic (Supplementary Table 8), and phenotypic data (Supplementary Table 5). G+C content of *P. marinistellae* (63%) is also closer to *Tropicimonas isoalkanivorans* (64.6%), the type species of *Tropicimonas*, than *Pseudoruegeria aquimaris* (66.7%), the type species of *Pseudoruegeria* (Supplementary Tables 2, 5), providing additional support for this reclassification.

Using a similar approach as above, we also propose the following reclassifications: the transfer of *Pelagivirga sediminicola* to the genus *Roseovarius* (Supplementary Figure 2A) and *Pelagibaca abyssi* to the genus *Salipiger* (Supplementary Figure 2B). It is important to note that the organisms we propose to merge into a single genus share several phenotypic traits with each other (Supplementary Table 5). Therefore, in addition to phylogenetic (Figure 2) and genomic evidence (Supplementary Table 8), there are also phenotypic traits supporting the suggested taxonomic modifications. Paraphyletic genera are one of the easiest issues to resolve as monophyly is one of the few universally agreed upon rules for taxon definition; therefore, the expected taxonomic change is clear (i.e., the conflicting isolate must be transferred to the primary genus). In addition, representatives typically share many phenotypic traits and have high genomic similarities. As a result, very limited modification is required to the genus description after the inclusion of the conflicting isolate.

### Reclassifications at the Genus Level: Addressing Polyphyletic Genera

Unlike paraphyletic genera, polyphyletic ones are generally more difficult to resolve, as this is done by either merging conflicting genera with existing ones or giving them novel genus designations. However, the lack of resolution from genomic similarity indicators, makes this task complicated. Following the polyphasic approach, if genomic similarity and phylogenetic analyses are inconclusive, the decision must then rely on phenotypic traits.

Based on our core-genome phylogenetic analysis, 17 polyphyletic genera were also identified (Figure 2 and Supplementary Table 7), 11 of which (*Albidovulum, Celeribacter, Defluviimonas, Gemmobacter, Lutimaribacter, Oceanicola, Primorskyibacter, Pseudorhodobacter, Rhodobacter, Roseivivax, Thalassobius*) (Supplementary Figures 3A–K) were confirmed by four recent studies (Arahal et al., 2019; Suresh et al., 2019; Hördt et al., 2020; Liu et al., 2020a). All isolates that are part of a polyphyletic genus but are not part of the primary clade (i.e., clade containing the type species of the genus) will be merged with existing genera or given a new genus designation. For each clade where genus level reclassification is required, within and between genera comparisons for all relevant genera were performed.

**FIGURE 5:**
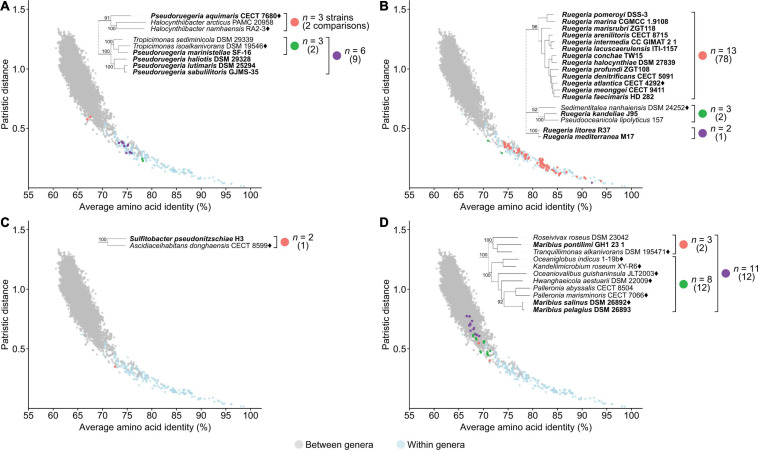
Dot plot for patristic distance (PD) against average amino acid identity (AAI) highlighting the comparisons of representative polyphyletic genera **(A)** *Pseudoruegeria*, **(B)** *Ruegeria*, **(C)** *Sulfitobacter*, **(D)** *Maribius*, and closely related organisms. PD and AAI comparisons for clades of interest are shown in orange, green, or purple. PD and AAI comparisons for all between (gray) or within (blue) recognized monophyletic genera are included as reference. The number of strains (*n*) is indicated for each clade of interest, and the number of relevant comparisons is shown in parentheses below. The corresponding phylogenetic trees are subsets of the core-genome tree (Figure 2). Names in bold are organisms of polyphyletic genera of interest. Diamonds after the names indicate the type species.

A majority of the comparisons between the polyphyletic genera and their closest neighbors resulted in borderline AAI and PD values, where they fall in the overlap region of between and within genus comparisons (Figure 5, Supplementary Figure 3, and Supplementary Table 8). Two genera (*Pseudoruegeria* and *Ruegeria*) could only be partially resolved based on phylogenetic and genomic data alone. Therefore, we turned to phenotypic data to fully resolve the inconsistencies. *Pseudoruegeria* is currently split into three clades (Figure 5A). The first clade contains the type species, *P. aquimaris*, and two *Halocynthiibacter* species. Genomic comparisons between *P. aquimaris* and the *Halocynthiibacter* species fall within the range of between genera comparisons. In addition, G+C content of *P. aquimaris* (66.7%) is also much higher from those observed among the two *Halocynthiibacter* species (52.8–53.2%) (Supplementary Table 5); therefore, both genera will retain their designations. The second clade contains *P. marinistellae*, which causes paraphyly of the *Tropicimonas* genus. This was resolved previously by the transfer of the species to *Tropicimonas*, as discussed above (Figure 4C). The third clade is composed exclusively of *Pseudoruegeria haliotis*, *Pseudoruegeria lutimaris*, and *Pseudoruegeria sabulilitoris*, with *Tropicimonas* as the closest relative. Genomic comparisons between these three *Pseudoruegeria* and *Tropicimonas* isolates resulted in inconclusive values for both AAI and PD (Figure 5A). However, *P. haliotis*, *P. lutimaris*, and *P. sabulilitoris* differ from the genus *Tropicimonas* (which now includes *P. marinistellae*) in a few phenotypic traits including growth at 45°C, growth at pH 5, and fatty acids and polar lipid contents (Supplementary Table 5). Since the type species *P. aquimaris* is not part of this clade, we propose to move the representatives of this group to a novel genus *Aliiruegeria* gen. nov., with *Aliiruegeria lutimaris* comb. nov. as the type species.

*Ruegeria* is currently split into three different clades (Figure 5B). The first clade contains the type species *Ruegeria atlantica* and is monophyletic with other *Ruegeria* isolates. This is the primary clade and will therefore retain the genus designation. The second clade contains *Ruegeria kandeliae*, *Sedimentitalea nanhaiensis*, and *Pseudooceanicola lipolyticus*. It is worth noting that *Pseudooceanicola* is also a polyphyletic genus with the type species placed elsewhere; therefore, like *R. kandeliae*, the genus designation of *P. lipolyticus* must also be reconsidered. Although genomic metrics between these three species are inconclusive, they differ in G+C content and several phenotypic characteristics including motility, Na^+^ requirement for growth, fatty acid content, growth at 4°C, and growth at pH > 9 (Supplementary Table 5); therefore, following the polyphasic approach, we suggest that these isolates remained as separate genera. Consistent with our analysis, *R. kandeliae* has recently been proposed to be transferred to a novel genus *Albibacillus* (Hördt et al., 2020). We then propose to rename *P. lipolyticus* to *Aliioceanicola lipolyticus* gen nov., comb. nov. The third clade is composed exclusively of *Ruegeria litorea* and *Ruegeria mediterranea* (Figure 5B) and will also be given a new genus designation, for which we propose the name *Falsiruegeria* gen. nov. and designate *Falsiruegeria litorea* comb. nov. as the type species.

The four remaining genera cannot be partially or fully resolved based solely on phylogenetic and genomic data; therefore, any reclassification relied more heavily on the examination of phenotypic traits. This includes *Sulfitobacter* (Figure 5C), *Maribius* (Figure 5D), *Ponticoccus* (Supplementary Figure 3L), and *Pseudooceanicola* (Supplementary Figure 3M).

*Sulfitobacter* is an example where phenotypic data clearly supports the splitting of the genus into multiple separate genera. *Sulfitobacter pseudonitzschiae* clusters separately from the primary *Sulfitobacter* clade containing the type species *Sulfitobacter pontiacus* (Figures 2, 5C). Since *S. pseudonitzschiae* differs from other *Sulfitobacter* in phenotypic traits including polar lipid contents and tolerance to various NaCl concentrations and pH levels, and its G+C content differs from the range observed among other *Sulfitobacter* (Supplementary Table 5), it is therefore appropriate to transfer it to a novel genus, which we propose to be named *Pseudosulfitobacter* gen. nov.

*Maribius* is an example where phenotypic data supports the merging of the non-primary clade (i.e., clade that does not contain the type species of the genus) with an existing genus. Currently, *Maribius* is split into two separate monophyletic clades (Figure 5D). One clade is composed of *Maribius salinus* (type species) and *Maribius pelagius*; this clade will retain the genus designation. *Maribius pontilimi* forms a monophyletic clade with *Tranquillimonas alkanivorans* and *Roseivivax roseus* and have several phenotypic traits in common, mainly in carbon utilization, differentiating them from other *Maribius* isolates (Supplementary Table 5). The polyphyletic genus *Roseivivax* (Supplementary Figure 3J), has *R. roseus* clustering separately from the primary *Roseivivax* clade and differing from other *Roseivivax* species in various phenotypic traits. We therefore propose to transfer *M. pontilimi* into the genus *Tranquillimonas*, as this is the first proposed genus in this group. Consistent with our analysis, *R. roseus* was recently transferred into the genus *Tranquillimonas* as *T. rosea* by Hördt et al. (2020).

Other polyphyletic genera were resolved following a similar approach. Overall, we identified 24 para- and polyphyletic genera based on our analyses (Supplementary Table 7). Those fully resolved from recent studies (Arahal et al., 2019; Suresh et al., 2019; Hördt et al., 2020; Liu et al., 2020a) (Figure 2) were not reclassified here again. All other newly proposed taxonomic changes from this study (described above) are listed in Figure 2 and Supplementary Table 9.

### Phylogenetic and Genomic Analyses Show a Clear Distinction Between the Two Major *Rhodobacteraceae* Lineages: Proposal to Move the Roseobacter Clade Into a New Family *Roseobacteraceae* fam. nov.

It is important to note that the roseobacter clade is not an official taxon name. In fact, there is no standardized terminology to refer to this clade. It was previously referred to as the marine roseobacter clade based on marine adaptations (Simon et al., 2017). However, as not all of the roseobacter clade live in marine environments and not all isolates outside of the roseobacter clade live in non-marine environments (Supplementary Table 2), this description does not distinguish the roseobacter clade specifically but rather a polyphyletic group within the *Rhodobacteraceae* family.

To establish a phylogenetically coherent classification for the roseobacter clade, we performed a meta-analysis of phenotypic traits as well as comprehensive genomics and phylogenomic analyses looking at similarities and differences between the roseobacter clade and its closest relatives. We identified several genomic and probable phenotypic differences between the roseobacter clade and the rest of *Rhodobacteraceae*. As such, we propose to move this clade to a new family, *Roseobacteraceae* fam. nov., based on the first described genus, *Roseobacter* (Shiba, 1991). All other species outside of this clade will remain as *Rhodobacteraceae*.

**FIGURE 6:**
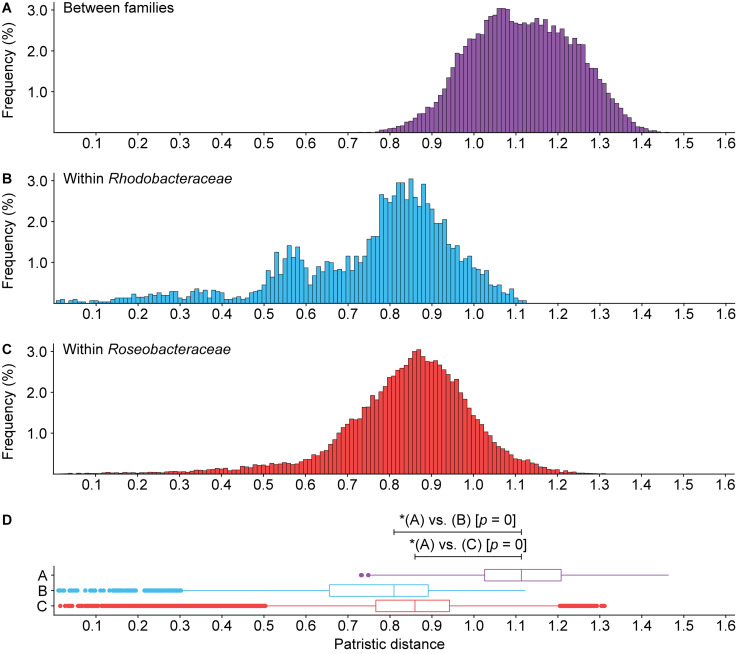
Histogram of patristic distance for comparisons **(A)** between *Roseobacteraceae* fam. nov. and *Rhodobacteraceae* (purple), **(B)** within *Rhodobacteraceae* only (blue), or **(C)** within *Roseobacteraceae* fam. nov. only (red). **(D)** Box plots show the 1.5 interquartile range, 25th, 50th, and 75th percentile. Asterisks (*) indicate significant differences between distributions (*p* < 0.05).

**FIGURE 7:**
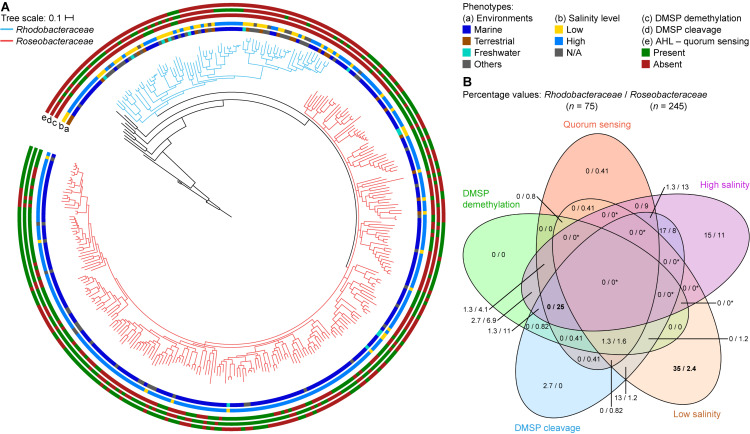
Differences between *Rhodobacteraceae* and *Roseobacteraceae* fam. nov. based on environment of isolation and phenotypic traits. **(A)** On the core-genome phylogenetic tree (same as Figure 2), branches of the families *Rhodobacteraceae* and *Roseobacteraceae* fam. nov. are highlighted in blue and pink, respectively. The scale bar represents amino acid substitutions per site. Rings represent the (a) environment of isolation – marine (blue), terrestrial (brown), freshwater (light blue), and others (gray); (b) salinity levels – high: ≥3.5% NaCl, light blue; low: <3.5% NaCl, yellow; and the presence (green) or absence (red) of (c) DMSP demethylation pathway, (d) DMSP cleavage pathway, and (e) AHL-quorum sensing. **(B)** Venn diagram showing percentage of isolates that are positive for each phenotypic trait considered. Since isolates are categorized to belong to either a high or a low salinity environment but never both, regions overlapping high and low salinity will always be 0/0 (and indicated by asterisks; otherwise, true 0/0 values are not). Numbers in bold highlight phenotype(s) most useful in distinguishing the two families (i.e., largest difference in percentage between the two families).

Phylogenetically, *Roseobacteraceae* fam. nov. is monophyletic with 100% bootstrap support, clearly separating it from *Rhodobacteraceae* (Figure 2 – inner ring). This is consistent with studies that the roseobacter clade is monophyletic and distinct from the rest of the family (Simon et al., 2017; Parks et al., 2018). PD for within family comparisons is significantly smaller than between family comparisons (*p* = 0) (Figure 6). Also, values of AAI (Supplementary Figure 4A) and CP similarities for within family comparisons are significantly higher than between family comparisons (*p* = 0) (Supplementary Figures 4B–D). Taken together, there is strong evidence that the *Roseobacteraceae* fam. nov. is phylogenetically and genomically distinct from *Rhodobacteraceae* and should be considered a novel family. The family classification we proposed here refer specifically to the two major phylogenetic clades of *Rhodobacteraceae* (Figure 7A). The remaining basal isolates (i.e., *Acuticoccus*, *Ahrensia*, *Albimonas*, *Amylibacter*, *Monaibacterium*, *Neptunicoccus*, *Oceanicella*, *Pontivivens*, and *Rubrimonas*) were excluded because they are monospecific, meaning only one named species for the genus is included in our phylogenetic tree either because only one species has been identified so far, no genome sequence is available for other species, or the other genomes did not meet our quality check standards (Supplementary Table 3). These basal isolates also do not form any distinguishable monophyletic clades and are perhaps erroneously placed in this order. In fact, one of the basal genus, *Acuticoccus*, has recently been proposed to belong to a novel family, *Acuticoccaceae* (Lai et al., 2019). For an accurate evaluation their taxonomic classifications, these basal isolates will not only require the inclusion of neighboring families but must also wait until additional strains or additional genomes for various monospecific genera become available.

### Predicted Phenotypic Characteristics Show Differences in Adaptive Traits Between the *Roseobacteraceae* fam. nov. and *Rhodobacteraceae*

Environmentally, the two families are different where 89% of the *Roseobacteraceae* fam. nov. are isolated in environments with high salt content (defined here as environments with ≥3.5% w/v NaCl, the average NaCl concentration of sea water) (Figure 7B, Supplementary Table 2, and Supplementary Figure 5), whereas only 39% of the *Rhodobacteraceae* family are isolated from such environments. This is consistent with a marine lifestyle for most of *Roseobacteraceae* fam. nov., a trait that was most certainly ancestral to this phylogenetic group with any exceptions representing derived traits (Buchan et al., 2005; Simon et al., 2017). Different environments also lead to different adaptions. Three pathways that are ancestral to *Roseobacteraceae* fam. nov. and characteristic of it without being universal were identified by combining a meta-analysis of phenotypic traits with comprehensive genomic similarity analyses. For each pathway, we chose functional marker genes as indication of presence/absence.

#### Sulfur Metabolism: DMSP Demethylation and DMSP Cleavage Pathways

Dimethylsulfoniopropionate is a ubiquitous sulfur containing compound found in the ocean produced by many marine phytoplankton and macroalgae, which can serve as an osmo- and cryoprotectant (Moran et al., 2012), antioxidant (Sunda et al., 2002), or as a defense mechanism against grazing (Strom et al., 2003). As DMSP is also a source of carbon and sulfur for marine bacteria, it is a known chemoattractant (Seymour et al., 2010). They can utilize DMSP in two ways (Moran et al., 2012): the demethylation pathway, which produces methanethiol (MeSH), and the cleavage pathway, which produces DMS (Moran et al., 2003; Todd et al., 2007; Reisch et al., 2011). MeSH is an important source of cellular sulfur and it has long been known that bacteria can incorporate MeSH directly into sulfur containing amino acids (Visscher et al., 1992; González et al., 1999). The second pathway cleaves DMSP into DMS, a volatile sulfur compound that plays an important role in global climate regulation (Lovelock et al., 1972; Charlson et al., 1987; Vallina and Simó, 2007; Moran et al., 2012) and is an important part of the sulfur cycle. *Roseobacteraceae* fam. nov. is one of the few organisms that is known to contain both pathways (Moran et al., 2003), suggesting the importance of DMSP to this family.

All isolates capable of DMSP demethylation have at least one homolog each of the *dmdABC* genes (Moran et al., 2012). These genes were therefore used as functional markers for the demethylation pathway, where only isolates with at least one homolog of each are potentially capable of DMSP demethylation. The cleavage pathway is more complicated, as there are six homologous DMSP lyases (*dddWPQDLY*) and not only can an isolate contain multiple copies of each gene, it is also not necessary to have all six homologs for a functional pathway (Moran et al., 2012). Therefore, isolates that contain any number of the six genes are considered to potentially be able to cleave DMSP. Overall, 64% of the *Roseobacteraceae* fam. nov. species are potentially capable of the cleavage pathway, which is significantly higher than the 37% of *Rhodobacteraceae* that are possibly able to do so (Figure 7B, Supplementary Table 10, and Supplementary Figure 5). The demethylation pathway shows a similar pattern where 52% of *Roseobacteraceae* fam. nov. are likely able to perform DMSP demethylation compared to only 7% of *Rhodobacteraceae*. Overall, 68% of *Roseobacteraceae* are capable of at least one of the two DMSP degradation pathways compared to only 41% of *Rhodobacteraceae* (Figure 7B and Supplementary Table 10). DMSP cleavage and DMSP demethylation are present in the majority of *Roseobacteraceae* fam. nov., suggesting that these two pathways are ancestral traits within this family and were subsequently lost by some. The importance of DMSP to *Roseobacteraceae* fam. nov. is further highlighted by the fact that 40% of them are likely capable of performing both pathways whereas only 3% of *Rhodobacteraceae* can. The difference in proportion of isolates capable of DMSP degradation between these two families makes biological sense, since the majority of *Roseobacteraceae* fam. nov. is found in the marine environment in association with marine algae blooms where DMSP is commonly found (Buchan et al., 2005).

#### Quorum Sensing: Acyl-Homoserine Lactone Production and Response

Marine bacteria can be broadly classified as free-living (can thrive on minimal nutrient) or patch-associated (able to exploit small nutrient rich patches) (Seymour et al., 2010). Patch-associated bacteria, such as *Roseobacteraceae* fam. nov., generally have a larger genome size encoding a variety of genes that allow these bacteria to respond quickly to changes in the environment (Luo and Moran, 2015). One of the adaptations *Roseobacteraceae* fam. nov. has is quorum sensing, an important behavioral modulation mechanism that regulates many phenotypes that requires coordinated behavior, such as biofilm formation and pathogenicity (Wagner-Döbler et al., 2005; Case et al., 2008). This mechanism allows bacteria to quickly respond in a population-dependent manner to different environmental cues and effectively cope with the changes in their environments.

Acyl-homoserine lactone-based quorum sensing (AHL-QS) is the most commonly described QS mechanism in *Proteobacteria* (Case et al., 2008) and is highly conserved within *Roseobacteraceae* fam. nov. (Cude and Buchan, 2013). A complete AHL-QS circuit consists of the *luxRI* genes (Case et al., 2008). The LuxR is the response regulator protein; it mediates gene expression of other proteins in the cell and activates the *luxI* gene. The LuxI is the synthase protein responsible for the synthesis of AHL. Not only can a single organism have more than one copy of the *luxRI* genes, there can also be more copies of the response regulator than the synthase (Case et al., 2008). In this study, isolates that contain at least one copy each of these genes are considered likely capable of AHL-QS. We found that 56% of *Roseobacteraceae* fam. nov. is potentially capable of AHL-QS, which is considerably higher than the 4% of *Rhodobacteraceae* (Figure 7B, Supplementary Table 10, and Supplementary Figure 5). Therefore, AHL-QS seems to be a trait that is more prominent in *Roseobacteraceae* fam. nov. than *Rhodobacteraceae*, likely because many live a patch-associated lifestyle in marine environments.

Examining all phenotypic traits together, we found that isolation from a high salinity environment together with the simultaneous ability to perform DMSP cleavage, DMSP demethylation, and quorum sensing may be strong indicators of the organism belonging to *Roseobacteraceae* fam. nov. In contrast, isolation from a low salinity environment together with a lack of quorum sensing ability may be a strong indicator of belonging to *Rhodobacteraceae* (Supplementary Table 10 and Supplementary Figure 6).

### Workflow for the Incorporation of New Genomes for Consistent Genus and Species Classifications

As it is not practical to reconstruct core-genome phylogenetic trees of all type strains each time new genomes become available, there needs to be a way to identify phylogenetic relationships of unknown isolates to known isolates quickly and accurately without solely relying on 16S rRNA gene phylogeny. AAI was used as a quick and efficient way to shorten the list of close relatives for the incorporation of new genomes. Ideally, the ten closest relatives can be determined based on pairwise AAI comparisons between the unknown isolate and all type strains. If the unknown isolate is proposed to belong to an existing genus, it is also important to include the type species and some, if not all, representatives of that genera (if these are not already part of the top ten isolates) to accurately determine the phylogenetic placement of the unknown isolate. This will significantly reduce the dataset from hundreds or even thousands of species to <20 species, for which in-depth phylogenomic analyses can readily be done.

We collected the genomes of 29 additional type strains that became available only after the commencement of this study (Supplementary Table 1). The identity of the 21 species were confirmed as they formed a strongly supported monophyletic clade with their proposed genera (Supplementary Figure 7). One of the confirmed species was *Primorskyibacter sedentarius*, the type species of the genus *Primorskyibacter*. This allowed us to resolve issues within this genus, which is currently split into two clades; one clade containing *Primorskyibacter sedentarius*, *Primorskyibacter marinus*, and *Pseudooceanicola flagellatus*, while the other contains *Primorskyibacter insulae* with the genus *Marivita* (Supplementary Figure 7A). As *Pseudooceanicola* is a polyphyletic genus with the primary species elsewhere in the tree, *P. flagellatus* must be renamed. The PD between *P. marinus* and *P. flagellatus* is 0.042, whereas the PD is 0.41 with its next closest relatives (*Puniceibacterium*). Similarly, AAI between *P. marinus* and *P. flagellatus* is 95% but is only 71% when compared with the two *Puniceibacterium* species (Supplementary Table 8 and Supplementary Figure 3G). This extremely high AAI value suggests that there is even the possibility that the two isolates (*P. marinus* and *P. flagellatus*) belong to the same species (Konstantinidis and Tiedje, 2005), but a dDDH value of 52.4% (Supplementary Table 8) clearly shows that these are different species. Based on genomic metrics alone, *P. marinus* is more closely related to *P. flagellatus* than its next closest relatives. In addition, *P. marinus* and *P. sedentarius* share several phenotypic traits with *P. flagellatus*, such as growth at 8% NaCl, growth at 40°C, G+C content, and oxidase and catalase activities (Supplementary Table 5). Taken together, we propose to transfer *P. flagellatus* to the genus *Primorskyibacter*. Consequently, *P. insulae* requires a novel genus designation, as it differs from the genus *Marivita* in a number of phenotypic traits as well, for which *Pseudoprimorksyibacter* was proposed recently (Hördt et al., 2020).

The identity of *Rhodobaca bogoriensis* is also confirmed as it forms a strongly supported monophyletic clade with the *Rhodobaca barguzinensis* (Supplementary Figure 7B). It was also immediately obvious from the phylogenetic tree that these two isolates are closely related as is evident by their short branch lengths. ANI and dDDH were therefore calculated for these two isolates, which were 100% for both. This suggests that *R. barguzinensis*, being described later, is part of the *R. bogoriensis* species. Not surprisingly, both organisms have many phenotypic traits in common such as their utilization of similar nitrogen and carbon sources, catalase activity, and sulfide utilization and resistance. They also have similar G+C content (59%). They do differ in some phenotypic traits including the utilization of a few carbon and nitrogen sources and resistance to a few antibiotics (Supplementary Table 5) (Boldareva et al., 2008). We therefore propose the reclassification of *R. barguzinensis* to *Rhodobaca bogoriensis* subsp. *barguzinensis* subsp. nov.

This approach was also able to highlight two misclassifications, the first of which is *Sinirhodobacter*, a novel genus proposed in 2013 as a close relative of *Rhodobacter* (Yang et al., 2013). Yang et al. (2013) have shown that *Sinirhodobacter* is the sister taxon of the genus *Rhodobacter*, with *Thioclava* being basal to both (Yang et al., 2013). This relationship was confirmed by core-genome phylogeny (Supplementary Figure 7C). However, different from the previous study, our updated analysis shows that *Paenirhodobacter* is a closer relative to *Sinirhodobacter* than *Rhodobacter*. In addition, *Paenirhodobacter* forms a monophyletic clade with *Sinirhodobacter populi*, resulting into paraphyly for *Sinirhodobacter*. *Paenirhodobacter* is likely misclassified as not only are both *Paenirhodobacter* and *Sinirhodobacter* differentiated from *Rhodobacter* by their lack of phototrophic abilities, the initial analyses describing *Paenirhodobacter* did not include any *Sinirhodobacter* strains (Yang et al., 2013; Wang D. et al., 2014). In addition, *Paenirhodobacter* also shares several phenotypic traits with *Sinirhodobacter* as both are positive for urease activity, arginine dihydrolase, and utilization of maltose but negative for indole production. Since *Paenirhodobacter* was described (Wang D. et al., 2014) after *Sinirhodobacter* (Yang et al., 2013), we propose to rename *Paenirhodobacter enshiensis* (currently the only named species of this genus) as *Sinirhodobacter enshiensis* comb. nov.

Processing newly available genomes of type strains also identified a second misclassification, that of *Phaeobacter marinintestinus*. Our core-genome phylogeny shows that the genus *Phaeobacter* is a monophyletic sister clade of *Ruegeria*, but *P. marinintestinus* is basal to both genera with 100% bootstrap support (Supplementary Figure 7D). *P. marinintestinus* was initially placed in *Phaeobacter* based solely on 16S rRNA gene and *gyrB* phylogenetic trees with bootstrap supports of 60.1% and 88%, respectively (Lee et al., 2015). In addition, a number of phenotypic differences exist between *P. marinintestinus* and other representatives of the genus, such as the ability to utilize and hydrolyze different carbon compounds as well as in various antibiotic resistance traits and enzymatic activities (Lee et al., 2015). Consequently, we propose to transfer *P. marinintestinus* to a novel genus *Falsiphaeobacter* gen. nov., where *Falsiphaeobacter marinintestinus* comb. nov. will be the type species.

There were also seven newly proposed genera for which the placements cannot be confirmed due to insufficient phylogenetic and genomic data, mostly as the result of a lack of representatives with full genome sequences. Lone species of *Rubellimicrobium* and *Falsirhodobacter* (*Rubellimicrobium roseum* and *Falsirhodobacter deserti*, respectively) are basal in the tree to their top ten closest relatives (Supplementary Figures 7E,F). There is currently insufficient information to make any conclusion regarding their taxonomic placement (i.e., whether they should remain in their current genera or be merged with neighboring isolates). It could be that with more data, these basal isolates will remain as the most basal strain and not cause any issue or eventually cause poly- or paraphyly. The genome of type species *Rubellimicrobium thermophilum* did not meet our quality threshold and was excluded from our analyses (Supplementary Table 3), whereas the genome of type species *Falsirhodobacter halotolerans* is currently not available. The remaining five genera (*Histidinibacterium*, *Aliishimia*, *Aquicoccus*, *Youngimonas*, and *Chachezhania*) are also not causing any issues of poly- or paraphyly (Supplementary Figures 7E,G–I) and reclassifications are not warranted.

Finally, the correct phylogenetic placement of newly available genomes for *Roseinatronobacter*, *Paracoccus*, *Rhodovulum*, and *Roseovarius* were also confirmed (Supplementary Figures 7B,J–M).

The examples presented here highlight the benefits of this approach as an efficient first step in determining identities of novel genomes as it can provide validation to the proposed taxonomic classification or illuminate potential misclassifications. However, its continued success is dependent on computing power growing as the number of genomes increases. If this becomes an issue, close relatives can still be identified using tools such as GTDB-tk (Chaumeil et al., 2019) to identify the general phylogenetic placements of unknown isolates among known type strains and allowing a shortlist of close relatives to be selected for more in-depth phylogenomic analyses. It should be noted that although phylogenetic data and AAI alone may not be sufficient to justify all taxonomic classifications, this approach can serve to guide subsequent in-depth genomic, phylogenetic, and phenotypic analyses that involve an even larger dataset of closely related strains.

## Conclusion

This study established a whole-genome-based phylogeny of *Rhodobacteraceae* type strains and incorporated various metrics – AAI, CP similarity, PD, dDDH, ANI – for pairwise genomic comparisons to fix taxonomic misclassifications within this group (e.g., para- and polyphyletic genera, species-level misidentifications). Additionally, *Roseobacteraceae* fam. nov. is a new family proposed in this study to split the roseobacter clade into its own family, which has been shown to be distinct from *Rhodobacteraceae* based on genomic, phylogenetic, and *in silico*-predicted phenotypic data. Lastly, this work successfully demonstrated a more efficient polyphasic approach to classifying newly sequenced isolates, as the reconstruction of a core-genome phylogenetic tree of all representatives is not practical each time a new genome becomes available. Overall, this work will serve as a foundation for the classification/reclassification of current and future *Rhodobacteraceae* and *Roseobacteraceae* fam. nov. isolates as more genomes become available for these continually and rapidly growing families of bacteria.

## Taxonomic Descriptions: New Family

### Description of *Roseobacteraceae* fam. nov.

Ro.se.o.bac.ter.a’ce.ae. N.L. masc. n. *Roseobacter*, type genus of the family; L. fem. pl. suff. *-aceae*, ending to denote a family; N.L. fem. pl. n. *Roseobacteraceae*, the *Roseobacter* family.

This family is circumscribed based on core-genome phylogeny. It is one of the most abundant groups of bacteria in marine ecosystems. It is phenotypically and metabolically diverse consisting of photoheterotrophic and chemoheterotrophic species. Many are of marine origin; some have been isolated from hypersaline and terrestrial environments. Many are isolated from high salt environments (i.e., ≥3.5% NaCl); many require NaCl for growth. Dimethylsulfoniopropionate degradation, either by the demethylation or cleavage pathway or both, is a common ability among species. Many also exhibit acyl-homoserine lactone-based quorum sensing. G+C content is 51.7–72.1%. The type genus is *Roseobacter* Shiba 1991.

## Taxonomic Descriptions: New Genera

### Description of *Aliioceanicola* gen. nov.

A.li.i.o.ce.a.ni’co.la. L. n. *alius*, other, another; L. n. *oceanus*, the ocean; L. masc. suff. *-cola*, inhabitant; L. masc. n. *Aliioceanicola*, another *Oceanicola*, referring to its original taxonomic classification as *Oceanicola*.

Cells are rod-shaped and are positive for oxidase and catalase. Cells require NaCl to grow and unable to grow at 4°C. The predominant ubiquinone is Q-10. The major fatty acids are 18:1 ω7c, 18:1 ω6c, 19:0 cyclo ω8c, 16:0 2-OH, and 16:0. The major polar lipids are phosphatidylcholine, phosphatidylethanolamine, and phosphatidylglycerol. G+C content is 64.6%. The type species is *Aliioceanicola lipolyticus*.

### Description of *Aliiruegeria* gen. nov.

Ali.i.rue.ge’ri.a. L. n. *alius*, other, another; M.L. fem. n. *Ruegeria*, a bacterial genus name honoring Rueger, a German microbiologist, for his contribution to the taxonomy of marine species; L. masc. n. *Aliiruegeria*, another *Ruegeria*, referring to its original taxonomic classification as *Pseudoruegeria*.

Cells are non-motile, rod, or ovoid shaped. The primary ubiquinone is Q-10. G+C content is 62.3–63%. Temperature range sustainable for growth is between 10 and 37°C. Growth is not observed at 45°C. Primary fatty acid content is 18:1 ω7c, 18:1 ω6c, 16:0, and 12:0 3-OH. Primary polar lipids are phosphatidylserine, phosphatidylcholine, phosphatidylglycerol, and phosphatidylethanolamine. Majority of the isolates are capable of nitrate reduction. The type species is *Aliiruegeria lutimaris*.

### Description of *Falsihalocynthiibacter* gen. nov.

Fal.si.ha.lo.cyn.thi.i.bac’ter. L. adj. *falsus*, false; N.L. fem. n. *Halocynthia*, genus name of the sea squirt; N.L. masc. n. *bacter*, rod; L. masc. n. *Falsihalocynthiibacter*, false *Halocynthiibacter*, referring to its original taxonomic classification as *Halocynthiibacter*.

Representatives of this genus are aerobic, non-motile, and non-gliding. Cells are rod shaped. Growth occurs at 10–27°C, pH between 5.5 and 9.5 and NaCl concentrations between 0.5 and 7.5% (w/v). G+C content is 53.2%. The type species is *Falsihalocynthiibacter arcticus*.

### Description of *Falsiphaeobacter* gen. nov.

Fal.si.phae.o.bac’ter. L. adj. *falsus*, false; Gr. adj. *phaeos*, dark, brown; N.L. masc. n. *bacter*, rod; L. masc. n. *Falsiphaeobacter*, false *Phaeobacter*, referring to its original taxonomic classification as *Phaeobacter*.

Cells are rod-shaped, non-motile, and strictly aerobic. Optimal growth occurs at 25°C, between pH 7–7.5 with 2–3% (w/v) NaCl. G+C content is 58.7%. The type species is *Falsiphaeobacter marinintestinus*.

### Description of *Falsiruegeria* gen. nov.

Fal.si.rue.ge’ri.a. L. adj. *falsus*, false; M.L. fem. n. *Ruegeria*, a bacterial genus name honoring Rueger, a German microbiologist, for his contribution to the taxonomy of marine species; L. masc. n. *Falsiruegeria*, false *Ruegeria*, referring to its original taxonomic classification as *Ruegeria*.

Cells are non-motile ovoid or rod-shaped and are positive for catalase and oxidase. G+C content is 58.9–59.2%. Cells are also positive for nitrate reduction and require Na^+^ for growth. The major fatty acids are 10:0 3-OH, 12:0 2-OH, 16:0, 16:0 2-OH, 18:0 iso, 18:1 ω6c, 18:0, 18:1 ω7c 11-methyl, 9:0, 12:0, 14:0, and 16:0. Temperature range for growth is 15–30°C. The type species is *Falsiruegeria mediterranea*.

### Description of *Pseudoponticoccus* gen. nov.

Pseu.do.pon.ti.coc’cus. Gr. masc. adj. *pseudês*, false; L. n. *pontus*, the sea; N.L. masc. n. *coccus*, berry; L. masc. n. *Pseudoponticoccus*, false *Ponticoccus*, referring to its original taxonomic classification as *Ponticoccus*.

Cells are nonmotile, ovoid and beige-brown in color. They are negative for nitrate reduction, growth at 2% (w/v) NaCl, and growth at pH 10. Cells are also negative for utilization of D-trehalose, D-cellobiose, gentiobiose, *N*-acetyl-D-glucosamine, *N*-acetyl-β-mannosamine, D-fructose, D-galactose, D-mannitol, D-arabitol, and glycerol. Growth occurs at temperatures between 15 and 40°C and pH between 5 and 9. G+C content is 68%. The type species is *Pseudoponticoccus marisrubri*.

### Description of *Pseudosulfitobacter* gen. nov.

Pseu.do.sul.fi.to.bac’ter. Gr. masc. adj. *pseudês*, false; M.L. n. *sulfitum*, sulfite; M. L. masc. n. *bacter*, equivalent to Gr. neut. n. *bacterion*, a rod; L. masc. n. *Pseudosulfitobacter*, false *Sulfitobacter*, referring to its original taxonomic classification as *Sulfitobacter*.

Cells are rod shaped. Temperature range for growth is 10–37°C and pH range for growth is 6–12. The predominant ubiquinone is Q-10. The major polar lipids are phosphatidylethanolamine and phosphatidylglycerol. G+C content 61.7%. The major fatty acids are 18:1 ω7c, 18:1 ω6c, and 16:0. The type species is *Pseudosulfitobacter pseudonitzschiae*.

## Taxonomic Descriptions: New (Combinations For) Species

### Description of *Aliioceanicola lipolyticus* comb. nov.

Basonym: *Pseudooceanicola lipolyticus* Hyun et al. 2013. The description is the same as that of *P. lipolyticus* (Huang et al., 2018). Genomic, phylogenetic, and phenotypic evidence strongly support the placement of this species in the genus *Aliioceanicola*. The type strain is 157^T^ (=KCTC 52654^T^ = MCCC 1K03317^T^). G+C content is 64.6%. The GenBank accession numbers for the type strain are PGTB01 (genome) and KY273603 (16S rRNA gene).

### Description of *Aliiruegeria haliotis* comb. nov.

Basonym: *Pseudoruegeria haliotis* Hyun et al., 2013. The description is the same as that of *P. haliotis* (Hyun et al., 2013). Genomic, phylogenetic, and phenotypic evidence strongly support the placement of this species in the genus *Aliiruegeria*. The type strain is DSM 29328^T^ (=JCM 18872^T^ = KACC 17214^T^ = WM67^T^). G+C content is 63%. The GenBank accession numbers for the type strain are PVTD01 (genome) and KC196070 (16S rRNA gene).

### Description of *Aliiruegeria lutimaris* comb. nov.

Basonym: *Pseudoruegeria lutimaris* Park et al. 2014. The description is the same as that of *P. lutimaris* (Jung et al., 2010). Genomic, phylogenetic, and phenotypic evidence strongly support the placement of the species in this genus *Aliiruegeria*. The type strain is DSM 25294^T^ (=CCUG 57754^T^ = HD-43^T^ = KCTC 22690^T^). G+C content is 62.3%. The GenBank accession numbers for the type strain are FNEK01 (genome) and FJ374173 (16S rRNA gene).

### Description of *Aliiruegeria sabulilitoris* comb. nov.

Basonym: *Pseudoruegeria sabulilitoris* Park et al. 2014. The description is the same as that of *P. sabulilitoris* (Park et al., 2014). Genomic, phylogenetic, and phenotypic evidence strongly support the placement of this species in the genus *Aliiruegeria*. The type strain is GJMS-35^T^ (=KCTC 42111^T^ = NBRC 110380^T^). G+C content is 62.4%. The GenBank accession numbers for the type strain are LOAS01 (genome) and KJ729032 (16S rRNA gene).

### Description of *Epibacterium horizontis* comb. nov.

Basonym: *Tritonibacter horizontis* Klotz et al. 2018. The description is the same as that of *T. horizontis* (Klotz et al., 2018). Genomic, phylogenetic, and phenotypic, evidence strongly support the placement of this species in the genus *Epibacterium*. The type strain is O3.65^T^ (=DSM 101689^T^ = LMG 29740^T^). G+C content is 61.5%. The GenBank accession number for the type strain is LPUY01 (genome and 16S rRNA gene).

### Description of *Falsihalocynthiibacter arcticus* comb. nov.

Basonym: *Halocynthiibacter arcticus* Baek et al. 2015. The description is the same as that of *H. arcticus* (Baek et al., 2015). Genomic, phylogenetic, and phenotypic evidence strongly support the placement of this species in the genus *Falsihalocynthiibacter*. The type strain is PAMC 20958^T^ (= JCM 30530^T^ = KCTC 42129^T^). G+C content is 53.2%. The GenBank accession numbers for the type strain are CP014327 (genome) and KP197665 (16S rRNA gene).

### Description of *Falsiphaeobacter marinintestinus* comb. nov.

Basonym: *Phaeobacter marinintestinus* Lee et al. 2015. The description is the same as that of *P. marinintestinus* (Lee et al., 2015). Genomic, phylogenetic, and phenotypic evidence strongly support the placement of this species in *Falsiphaeobacter* as it is unambiguously different from other *Phaeobacter* isolates. The type strain is UB-M7^T^ (= JCM 19926^T^ = KCCM 43045^T^). G+C content is 58.7%. The GenBank accession numbers for the type strain are VOGO01 (genome) and KJ461690 (16S rRNA gene).

### Description of *Falsiruegeria litorea* comb. nov.

Basonym: *Ruegeria litorea* (Lucena et al. 2014) Wirth and Whitman 2018. The description is same as that of *R. litorea* (Wirth and Whitman, 2018). Genomic, phylogenetic, and phenotypic data strongly support the placement of this species in the genus *Falsiruegeria*. The type strain is R37^T^ (=CECT 7639^T^ = KCTC 23353^T^). G+C content is 59.2%. The GenBank accession numbers for the type strain are FWFO01 (genome) and HE860713 (16S rRNA gene).

### Description of *Falsiruegeria mediterranea* comb. nov.

Basonym: *Ruegeria mediterranea* (Lucena et al. 2014) Wirth and Whitman, 2018. The description is same as the same as that of *R. mediterranea* (Wirth and Whitman, 2018). Genomic, phylogenetic, and phenotypic evidence strongly support the placement of this species in the genus *Falsiruegeria*. The type strain is M17^T^ (=CECT 7615^T^ = KCTC 23058^T^). G+C content is 58.9%. The GenBank accession numbers for the type strain are ONZG01 (genome) and HE860710 (16S rRNA gene).

### Description of *Primorskyibacter flagellatus* comb. nov

Basonym: *Pseudooceanicola flagellatus* (Huo et al. 2014) Huang et al. 2018. The description is the same as that of *Pseudooceanicola flagellatus* (Huang et al., 2018). Genomic, phylogenetic, and phenotypic evidence strongly support the placement of this species in the genus *Primorskyibacter*. The type strain is CGMCC 1.12644^T^ (=CGMCC 1.12664^T^ = DY470^T^ = LMG 27871^T^). G+C content is 60%. The GenBank accession numbers for the type strain are FWYD01 (genome) and KF434118 (16S rRNA gene).

### Description of *Pseudoponticoccus marisrubri* comb. nov.

Basonym: *Ponticoccus marisrubri* Zhang et al. 2017. The description is the same as that of *Ponticoccus marisrubri* (Zhang G. et al., 2017). Genomic, phylogenetic, and phenotypic evidence strongly support the placement of this species in the genus *Pseudoponticoccus*. The type strain is SJ5A-1^T^ (=ACCC19863^T^ = JCM 19520^T^). G+C content is 68%. The GenBank accession numbers for the type strain are LPXO01 (genome) and KP726358 (16S rRNA gene).

### Description of *Pseudosulfitobacter pseudonitzschiae* comb. nov.

Basonym: *Sulfitobacter pseudonitzschiae* Hong et al. 2015. The description is the same as that of *S. pseudonitzschiae* (Hong et al., 2015). Genomic, phylogenetic, and phenotypic evidence strongly support the placement of this species in the genus *Pseudosulfitobacter*. The type strain is H3^T^ (= DSM 26824^T^ = MCCC 1A00686^T^). G+C content is 61.7%. The GenBank accession numbers for the type strain are JAMD01 (genome) and KF006321 (16S rRNA gene).

### Description of *Roseovarius sediminicola* comb. nov.

Basonym: *Pelagivirga sediminicola* Ji et al. 2018. The description is the same as that of *P. sediminicola* (Ji et al., 2018). Genomic, phylogenetic, and phenotypic evidence strongly support the placement of this species in the genus *Roseovarius*. The type strain is Bh-SD19^T^ (= CCTCC AB 2017074^T^ = KCTC 62202^T^). G+C content is 63.9%. The GenBank accession numbers for the type strain are QCYH01 (genome) and MG775052 (16S rRNA gene).

### Description of *Salipiger abyssi* comb. nov.

Basonym: *Pelagibaca abyssi* Lin et al. 2014. The description is the same as that of *P. abyssi* (Lin et al., 2014). Genomic, phylogenetic, and phenotypic evidence strongly support the placement of this species in the genus *Salipiger*. The type strain is JLT2014^T^ (= CGMCC 1.12376^T^ = JL2014^T^ = LMG 27363^T^). G+C content is 65.9%. The GenBank accession numbers for the type strain are CP015093 (genome) and JX878396 (16S rRNA gene).

### Description of *Sinirhodobacter enshiensis* comb. nov.

Basonym: *Paenirhodobacter enshiensis* Wang et al. 2014. The description is the same as that of *P. enshiensis* (Wang D. et al., 2014). Genomic, phylogenetic, and phenotypic evidence strongly support the placement of this species in the genus *Sinirhodobacter*. The type strain is DW2-9^T^ (= CCTCC AB 2011145^T^ = KCTC 15169^T^). G+C content is 66.8%. The GenBank accession numbers for the type strain are JFZB01 (genome) and JN797511 (16S rRNA gene).

### Description of *Tranquillimonas pontilimi* comb. nov.

Basonym: *Maribius pontilimi* Lee 2018. The description is the same as that of *M. pontilimi* (Lee, 2018). Genomic, phylogenetic, and phenotypic evidence strongly support the placement of this species in the genus *Tranquillimonas*. The type strain is GH1-23^T^ (= DSM 104950^T^ = KCTC 52957^T^). G+C content is 68.1%. The GenBank accession numbers for the type strain are JAEKPD01 (genome) and LT797154 (16S rRNA gene).

### Description of *Tropicibacter varians* comb. nov.

Basonym: *Pelagimonas varians* Hahnke et al. 2013. The description is the same as that of *P. varians* (Hahnke et al., 2013). Genomic, phylogenetic, and phenotypic evidence strongly support the placement of this species in the genus *Tropicibacter*. The type strain is DSM 23678^T^ (= CIP 110297^T^ = LMG 26343^T^ = SH4-1^T^). G+C content is 55.2%. The GenBank accession numbers for the type strain are QKMF01 (genome) and FJ882053 (16S rRNA gene).

### Description of *Tropicimonas marinistellae* comb. nov.

Basonym: *Pseudoruegeria marinistellae* Zhang et al. 2017. The description is the same as that of *P. marinistellae* (Zhang Y. et al., 2017). Genomic, phylogenetic, and phenotypic evidence strongly support the placement of this species in the genus *Tropicimonas*. The type strain is SF-16^T^ (= KCTC 42910^T^ = MCCC 1K01155^T^). G+C content is 63%. The GenBank accession numbers for the type strain are LNCI01 (genome) and KT944035 (16S rRNA gene).

## Taxonomic Descriptions: New Subspecies

### Description of *Rhodovulum kholense* subsp. *viride* subsp. nov.

Vi’ri.de. L. neut. adj. *viride*, green, referring to a green bacterium.

Basonym: *Rhodovulum viride* Srinivas et al. 2014. The description is that same as that of *R. viride* (Srinivas et al., 2014) with the following modifications. G+C content is 67.6%. The type strain is JA756^T^ (= KCTC 15223^T^ = NBRC 109122^T^). The GenBank accession numbers for the type strain are MUAV01 (genome) and HE983843 (16S rRNA gene).

### Description of *Rhodobaca bogoriensis* subsp. *barguzinensis* subsp. nov.

Bar.gu.zin.en’sis. N.L. masc./fem. adj. *barguzinensis*, pertaining to Barguzin Valley, Russia, from where the type strain was isolated.

Basonym: *Rhodobaca barguzinensis* Boldareva et al. 2009. The description is the same as that of *R. barguzinensis* (Boldareva et al., 2008) with the following modifications. G+C content is 59%. The type strain is alga05^T^ (= DSM 19920^T^ = VKM B-2406^T^). The GenBank accession numbers for the type strain are CP024899 (genome) and EF554833 (16S rRNA gene).

## Taxonomic Descriptions: Emendations

### Emended Description of *Rhodobacteraceae* Garrity et al. 2006

The description is the same as before (Garrity et al., 2015b) with modifications. Species isolated from marine, terrestrial, and freshwater habitats. Many are isolated form low salt environments (i.e., <3.5% NaCl); many do not require NaCl for growth. Many are not capable of dimethylsulfoniopropionate degradation nor acyl-homoserine lactone-based quorum sensing. G+C content is 48.1–72.9%.

### Emended Description of *Epibacterium* Penesyan et al. 2013

The description is the same as before (Wirth and Whitman, 2018) with modification following the inclusion of *Epibacterium horizontis* (formerly known as *Tritonibacter horizontis*). NaCl range for growth is from 0 to 15% (w/v). G+C content is 53–60.9%.

### Emended Description of *Maribius* Choi et al. 2007

The description of is the same as before (Choi et al., 2007) with modifications following the exclusion of *Tranquillimonas pontilimi* (formerly known as *Maribius pontilimi*). Growth is not observed at 35°C or at 9% (w/v) NaCl. Representatives are negative for the utilization of D-galactose, D-glucose, lactose, maltose, melezitose, and L-rhamnose. It is negative for α-galactosidase, valine arylamidase, and β-glucosidase. G+C content is 66.9–67.7%.

### Emended Description of *Pelagimonas* Hahnke et al. 2013

Following the exclusion of *Tropicibacter varians* (formerly known as *Pelagimonas varians*), the previous type species of the genus (Hahnke et al., 2013), the new type species will be *Pelagimonas phthalicica*, currently the only species of this genus. The description of *Pelagimonas* will therefore follow the description of *P. phthalicica* (Hördt et al., 2020).

### Emended Description of *Pelagivirga* Ji et al. 2018

Following the exclusion of *Roseovarius sediminicola* (formerly known as *Pelagivirga sediminicola*), the previous type species (Ji et al., 2018), currently the only species of this genus is *Pelagivirga dicentrarchi*. The description of *Pelagivirga* therefore follows from the description of *Pelagivirga dicentrarchi* (Li et al., 2020). *Pelagivirga dicentrarchi* also becomes the new type species of this genus.

### Emended Description of *Ponticoccus* Hwang and Cho 2008

Description is the same as before (Hwang and Cho, 2008) with modifications following the exclusion of *Pseudoponticoccus marisrubri* (formerly known as *Ponticoccus marisrubri*). Growth occurs at NaCl concentration between 1 and 15% (w/v) and pH range from 5 to 10. G+C content is 67.4%. It is positive for the utilization of D-trehalose, D-cellobiose, gentiobiose, *N*-acetyl-D-glucosamine, *N*-acetyl-β-mannosamine, D-fructose, D-serine, D-Mannitol, D-arabitol and myo-Inositol and negative for D-mannose and glycerol. It is also positive for naphthol-AS-BI-phosphohydrolase and nitrate reduction.

### Emended Description of *Primorskyibacter* Romanenko et al. 2011

The description is the same as before (Romanenko et al., 2011) with modification following the inclusion of *Primorskyibacter flagellatus* (formerly known as *Pseudooceanicola flagellatus*). Cells are capable of growth at pH 5.5–9.5. G+C content is 60–60.8%.

### Emended Description of *Pseudooceanicola* Lai et al. 2015

The description is the same as before (Lai et al., 2015) with modifications following the exclusion of *Primorskyibacter flagellatus* (formerly known as *Pseudooceanicola flagellatus*) and *Aliioceanicola lipolyticus* (formerly known as *Pseudooceanicola lipolyticus*). Growth occurs at pH range between 6.5 and 9.5 and NaCl concentration between 0.5 and 8% (w/v). The major fatty acids are 18:1 ω7c, 11-methyl 18:1 ω7c, 16:0, 17:0, 18:0, 12:1 3-OH. G+C content is 64.1–67.9%.

### Emended Description of *Pseudoruegeria* Yoon et al. 2007

The description is the same as before (Yoon et al., 2007) with modifications following the exclusion of *Aliiruegeria lutimaris* (formerly known as *Pseudoruegeria lutimaris*), *Aliiruegeria haliotis* (formerly known as *Pseudoruegeria haliotis*), *Tropicimonas marinistellae* (formerly known as *Pseudoruegeria marinistellae*), and *Aliiruegeria sabulilitoris* (formerly known as *Pseudoruegeria sabulilitoris*). Growth occurs at temperature range between 15 and 49°C, pH range between 5.5 and 8 and NaCl concentration at most 8% (w/v). Growth is not observed at 0% (w/v) NaCl concentration. G+C content is 66.7%.

### Emended Description of *Roseovarius* Labrenz et al. 1999

The description is the same as before (Labrenz et al., 1999) with modifications following the inclusion of *Roseovarius sediminicola* (formerly known as *Pelagivirga sediminicola*). The primary fatty acids are C18:1, C18:2, C12:0 2-OH, C12:1 3-OH, C16:1, C16:0, C18:0, C18:1 ω7C, C18:1 ω7C 11-methyl, C10:0 3-OH, C16:0 2-OH, C10:0, C12:0, C17:0, C18:0 iso, C18:0 ω8C cyclo, C18:1 ω6C, C18:1 ω7c, C18:1 ω6c,16:1 ω9c, C18:0 methyl, C18:1 ω7 methyl, C19:1 cyclo, C19:0 ω8c, C18:1 ω7c 11-methyl, C18:1 ω9t, C18:1 ω12t. G+C content is 54.3–63.9%.

### Emended Description of *Salipiger* Martínez-Cánovas et al. 2004

The description is the same as before (Martínez-Cánovas et al., 2004) with modification following the inclusion of *Salipiger abyssi* (formerly known as *Pelagibaca abyssi*). G+C content range is 64.3–67.3%.

### Emended Description of *Sinirhodobacter* Yang et al. 2018

The description is the same as before (Yang et al., 2013) with modifications following the inclusion of *Sinirhodobacter enshiensis* (formerly known as *Paenirhodobacter enshiensis*). Cells have variable phenotypes for catalase and oxidase activity. The major fatty acids are 18:1 ω6c, 18:1 ω7c, 16:0, 18:0, 19:0 cyclo ω8c, iso-15:0 2-OH, 16:1 ω6c, and 16:1 ω7c. Some contain bacteriochlorophyll *a*. Temperature range for growth is 4–42°C. G+C content is 65–68%.

### Emended Description of *Sulfitobacter* Sorokin 1996

The description is the same as before (Sorokin, 1995) with modifications following the exclusion of *Pseudosulfitobacter pseudonitzschiae* (formerly known as *Sulfitobacter pseudonitzschiae*).

Growth is observed in pH range 5–11 and NaCl concentration 1–12% (w/v). Growth is not observed at pH of 12. Growth is observed for some at NaCl concentrations ≥ 10% (w/v) or at temperature less than 4°C. G+C content is 55.2–60.8%.

### Emended Description of *Tranquillimonas* Harwati et al. 2008

The description is the same as before (Harwati et al., 2008) with modification following the inclusion of *Tranquillimonas pontilimi* (formerly known as *Maribius pontilimi*). Representatives have variable phenotypes for valine arylamidase and α-galactosidase activity. G+C content is 67.3–68.1%.

### Emended Description of *Tritonibacter* Klotz et al. 2018

The description is the same as before (Hördt et al., 2020) with modifications following the exclusion of *Epibacterium horizontis* (formerly known as *Tritonibacter horizontis*), the previous type species. The new type species is *Tritonibacter scottomollicae*, the earliest named species of the genus. Growth occurs at 4–40°C. The major fatty acids are C10:0 3-OH, 11-methyl C18:1 ω7c, C16:0, C16:0 2-OH, C18:1 2-OH ω7c, 18:1 ω7c, 16:0, 18:2, 10:0 3-OH, 12:0, 20:1 2-OH, 18:0, C18:1 ω7c, 18:1 ω6c, C10: 3-OH.

### Emended Description of *Tropicibacter* Harwati et al. 2009

The description is the same as before (Harwati et al., 2009a) with modifications following the inclusion of *Tropicibacter varians* (formerly known as *Pelagimonas varians*). Representatives can be motile or non-motile. They can be positive or negative for catalase activity. The primary fatty acids are 18:1 ω7c, 16:0, 11 methyl 18:1 ω7c, 18:1 ω9c, 20:1 ω7c, 12:1 3-OH, 11 Methyl 18:1 ω7c, 10:0 3-OH, 12:1, 14:1 3-OH, 18:0, 11-methyl 18:1 ω7c. Growth can be observed between 4 and 43°C and pH range from 6 to 9.5. G+C content is 57.9–63.2%.

### Emended Description of *Tropicimonas* Harwati et al. 2009

The description is same as before (Harwati et al., 2009b) with modifications following the inclusion of *Tropicimonas marinistellae* (formerly known as *Pseudoruegeria marinistellae*). Cells are aerobic or facultative anaerobic. NaCl range for growth is 0–12%. G+C content is 63–66.4%.

## Data Availability Statement

The datasets presented in this study can be found in online repositories. The names of the repository/repositories and accession number(s) can be found in the article/Supplementary Material.

## Author Contributions

KL, FO, YB, and RC conceived the project. KL and FO carried out the phylogenetic and comparative genomic analyses. KL and FO drafted the original manuscript. FO, YB, and RC supervised the work. All authors edited, reviewed, and approved the manuscript.

## Conflict of Interest

The authors declare that this study received funding from the Bank of Montréal Financial Group. The funder was not involved in the study design, collection, analysis, interpretation of data, the writing of this article, or the decision to submit it for publication.
