# Supplementary material for: Roseobacters in a Sea of Poly- and Paraphyly: Whole Genome-Based Taxonomy of the Family Rhodobacteraceae and the Proposal for the Split of the “Roseobacter Clade” Into a Novel Family, Roseobacteraceae fam. nov
Source: Front Microbiol. 2021 Jun 25;12:683109. doi: 10.3389/fmicb.2021.683109 (PMC8267831; doi:10.3389/fmicb.2021.683109)
Supplement: Supplementary file 1 [file Data_Sheet_1.pdf]

Supplementary Material

Roseobacters in a Sea of Poly- and Paraphyly: Whole Genome-Based Taxonomy of the Family *Rhodobacteraceae* and the Proposal for the Split of the "Roseobacter Clade" Into a Novel Family, *Roseobacteraceae* fam. nov.

Kevin Y. H. Liang†, Fabini D. Orata†*, Yann F. Boucher, Rebecca J. Case*

*Correspondence: Fabini D. Orata, fabini.orata@ualberta.ca; Rebecca J. Case, rj.case@ntu.edu.sg

†These authors have contributed equally to this work and share first authorship.

1. Supplementary Tables#

Supplementary Table 1. All *Rhodobacteraceae* isolates used in this study including the low-quality genomes subsequently removed based on CheckM results.

Supplementary Table 2. Meta-information for all 331 high-quality genomes used in this study together with NCBI genome accession numbers.

Supplementary Table 3. CheckM results based on quality check criteria of ≥95% completeness and ≤5% contamination.

Supplementary Table 4. Genomic metrics for comparisons within or between recognized monophyletic genera.

Supplementary Table 5. Phenotypic data for organisms involved in species-level reclassifications (Sp_), as well as para- (Para_) and polyphyletic (Poly_) genera together with their closest neighbors. Number in parentheses indicate the number of isolates. ND, not determined.

Supplementary Table 6. Genomic metrics for comparisons within or between non-monospecific genera, as well as comparisons within or between families.

Supplementary Table 7. List of all genera sorted by status (i.e., monophyletic, paraphyletic, or polyphyletic).

Supplementary Table 8. Genomic metrics for all poly- and paraphyletic genera.

Supplementary Table 9. All genus and species level taxonomic changes proposed in this study.

Supplementary Table 10. *In silico* phenotypic analyses of *Rhodobacteraceae* and *Roseobacteraceae* fam. nov. Proportion Z-test was used to test significance of difference in proportion of genes present between *Rhodobacteraceae* and *Roseobacteraceae* fam. nov.

#*See separate Excel sheets for complete supplementary tables.*

2. Supplementary Figures

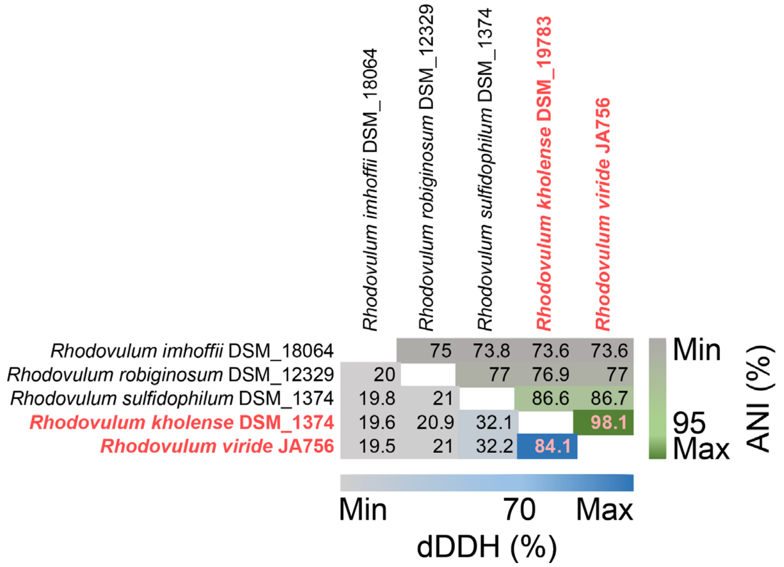

Supplementary Figure 1. Digital DNA–DNA hybridization (dDDH) and average nucleotide identity (ANI) values between *Rhodovulum* species. Species highlighted in red indicate conflict (i.e., they belong to the same species since the dDDH and ANI values above the species thresholds). The species thresholds (70% dDDH and 95% ANI) are labeled on the x- and y-axes, respectively.

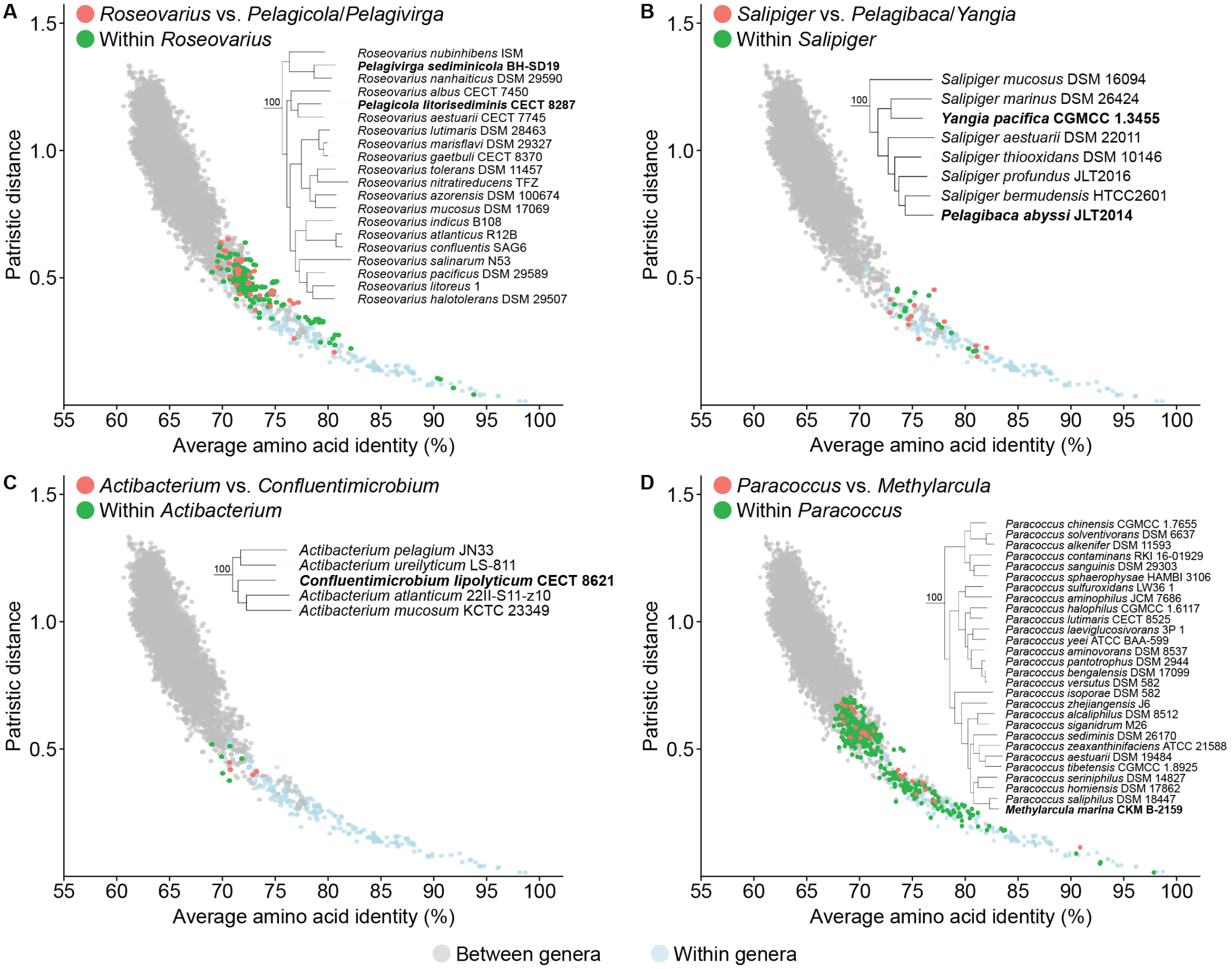

Supplementary Figure 2. Dot plot for patristic distance (PD) against average amino acid identity (AAI) highlighting the comparisons of paraphyletic genera (A) *Roseovarius*, (B) *Salipiger*, (C) *Actibacterium*, (D) *Paracoccus*, and closely related organisms. PD and AAI comparisons for between (orange) or within (green) genera of interest are shown. PD and AAI comparisons for all between (grey) or within (blue) recognized monophyletic genera are included as reference. The corresponding phylogenetic trees are subsets of the core-genome tree (Figure 2). Names in bold are organisms causing paraphyly of genera of interest.

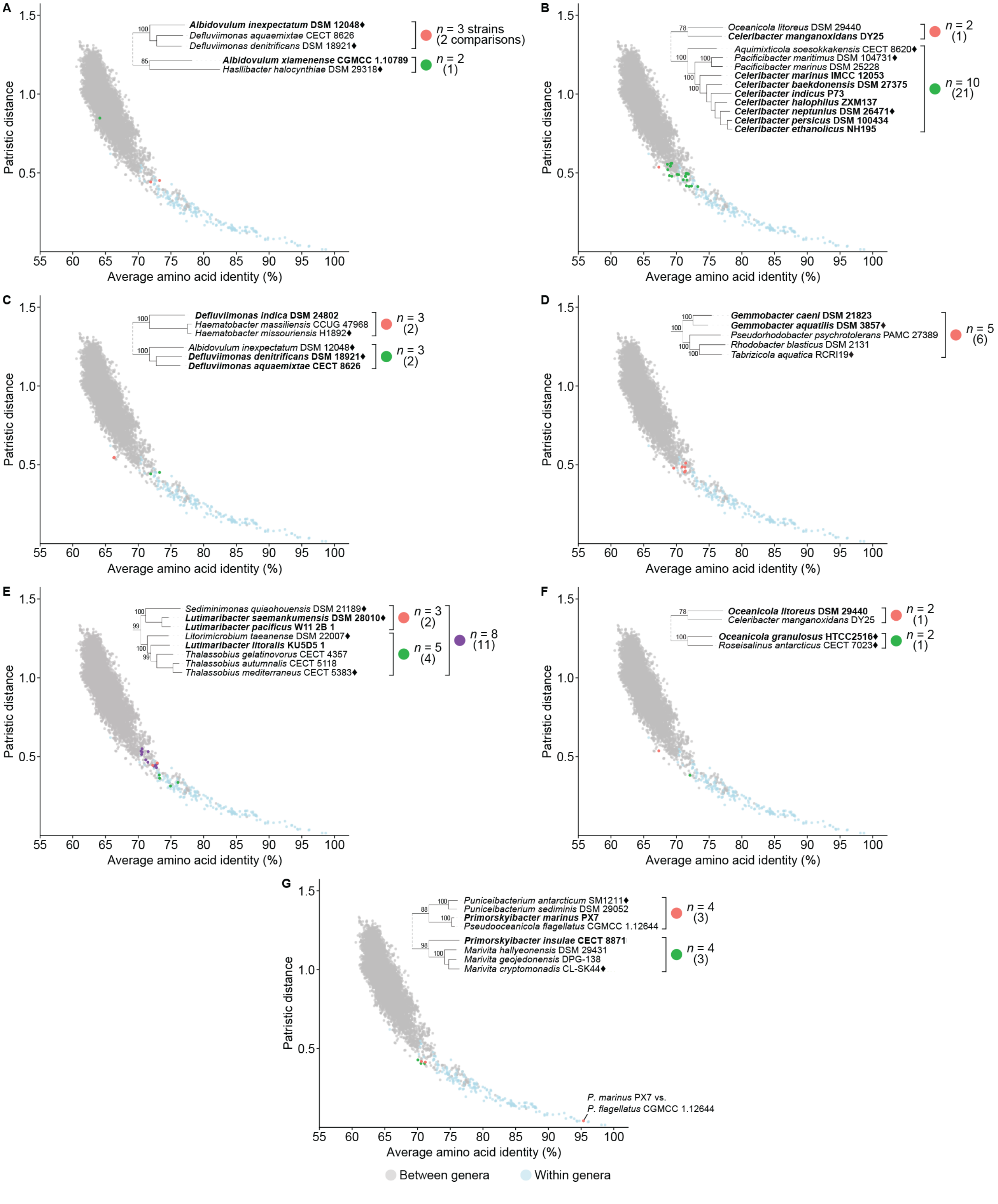

Supplementary Figure 3. (See legend on next page)

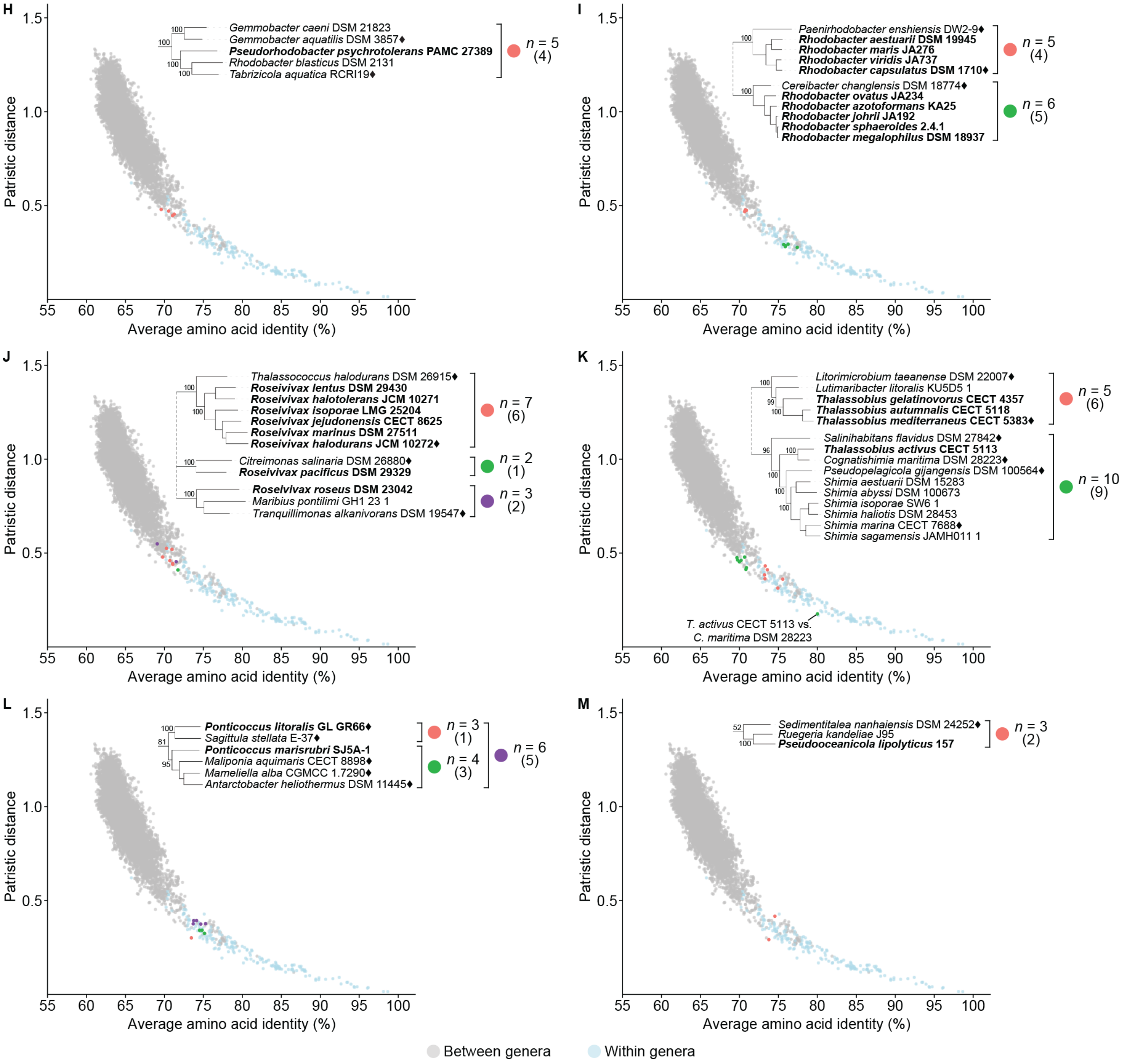

Supplementary Figure 3. Dot plot for patristic distance (PD) against average amino acid identity (AAI) highlighting the comparisons of polyphyletic genera (A) *Albidovulum*, (B) *Celeribacter*, (C) *Defluviimonas*, (D) *Gemmobacter*, (E) *Lutimaribacter*, (F) *Oceanicola*, (G) *Primorskyibacter*, (H) *Pseudorhodobacter*, (I) *Rhodobacter*, (J) *Roseivivax*, (K) *Thalassobius*, (L) *Ponticoccus*, (M) *Pseudooceanicola*, and closely related organisms. PD and AAI comparisons for clades of interest are shown in orange, green, or purple. PD and AAI comparisons for all between (grey) or within (blue) recognized monophyletic genera are included as reference. The number of strains (*n*) is indicated for each clade of interest, and the number of relevant comparisons is shown in parentheses below. The corresponding phylogenetic trees are subsets of the core-genome tree (Figure 2). Names in bold are organisms of polyphyletic genera of interest. Diamonds after the names indicate the type species.

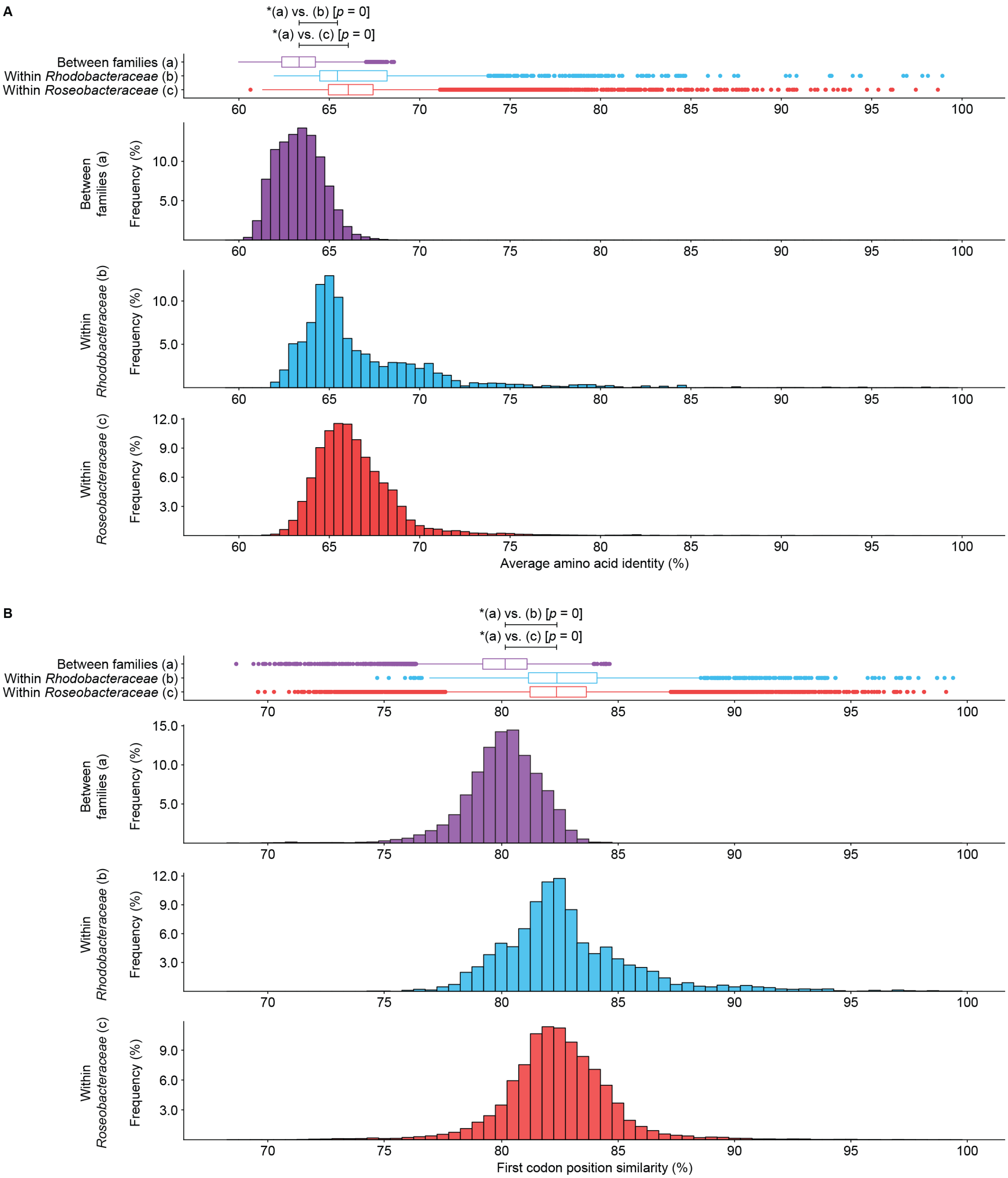

Supplementary Figure 4. (See legend on next page)

C

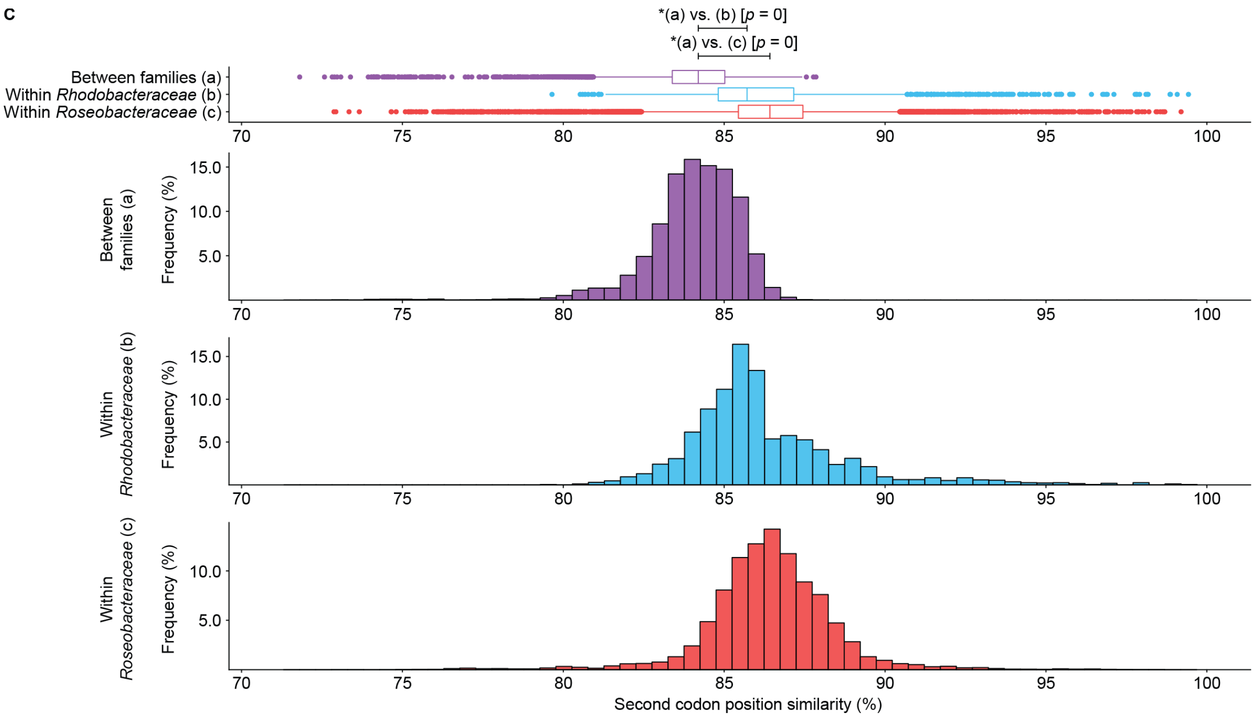

D

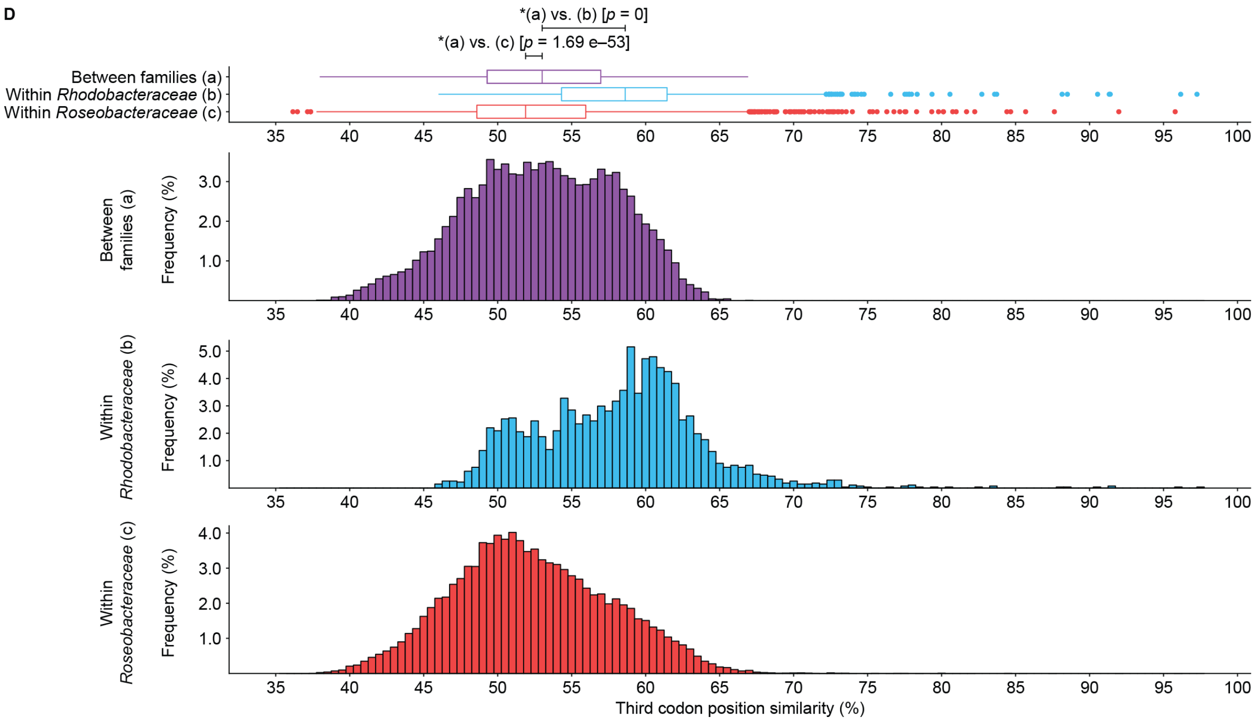

Supplementary Figure 4. Histogram of (A) average amino acid identity, as well as the (B) 1st, (C) 2nd, and (D) 3rd codon position similarities for comparisons between *Roseobacteraceae* fam. nov. and *Rhodobacteraceae* (a) (purple), within *Rhodobacteraceae* only (b) (blue), or within *Roseobacteraceae* fam. nov. only (c) (red). Box plots (top) show the 1.5 interquartile range, 25th, 50th, and 75th percentile. Asterisks (*) indicate significant differences between distributions ($p < 0.05$).

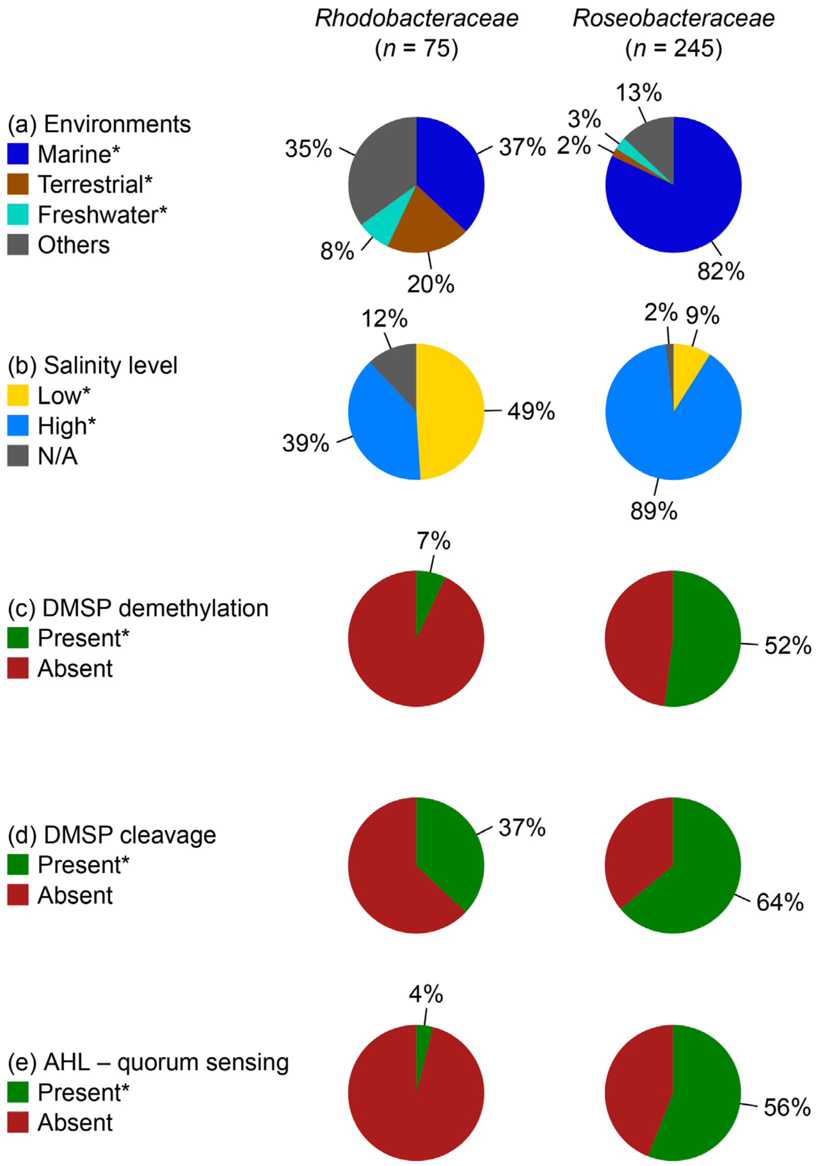

Supplementary Figure 5. Differences between *Rhodobacteraceae* and *Roseobacteraceae* fam. nov. based on environment of isolation and phenotypic traits. Phenotypes are the same as in Figure 7A – (a) environment of isolation – marine (blue), terrestrial (brown), freshwater (light blue), and others (grey); (b) salinity levels – high, ≥3.5% NaCl, light blue; low, <3.5% NaCl, yellow; and the presence (green) or absence (red) of (c) DMSP demethylation pathway, (d) DMSP cleavage pathway, and (e) AHL-quorum sensing. Asterisks (*) indicate traits with significant difference in proportion between the two families ($p < 0.05$ according to proportional Z-test).

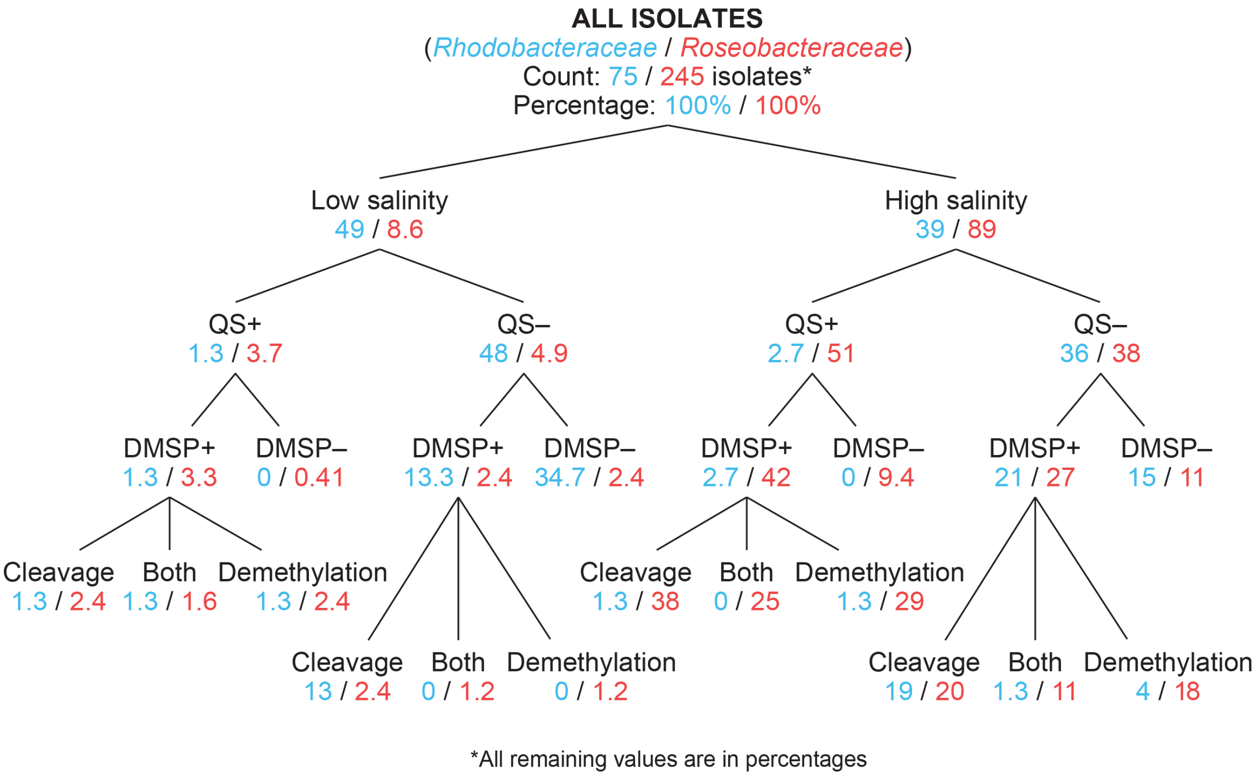

Supplementary Figure 6. Phenotype classification tree for all 75 representatives of *Rhodobacteraceae* and 245 representatives of *Roseobacteraceae* fam. nov. used in this study. Isolates outside of the two families (i.e., two major lineages) are omitted. Numbers are percentage of the total number of isolates (blue, *Rhodobacteraceae*; red, *Roseobacteraceae* fam. nov.) positive for each respective phenotype. QS, quorum sensing; DMSP, dimethylsulfoniopropionate; low salinity, <3.5% NaCl; high salinity, ≥3.5% NaCl; DMSP+, positive for DMSP demethylation, cleavage, or both; DMSP–, negative for DMSP demethylation and cleavage.

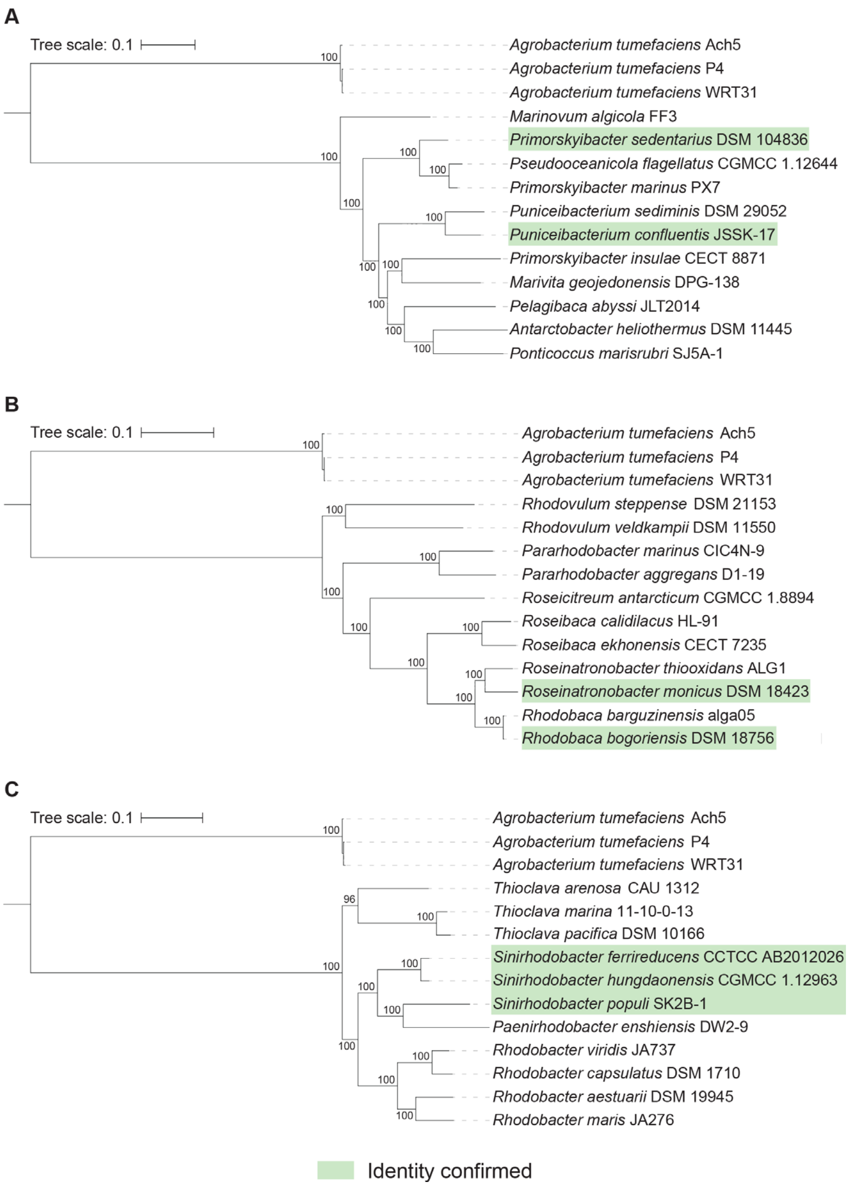

Supplementary Figure 7. (See legend on page 15)

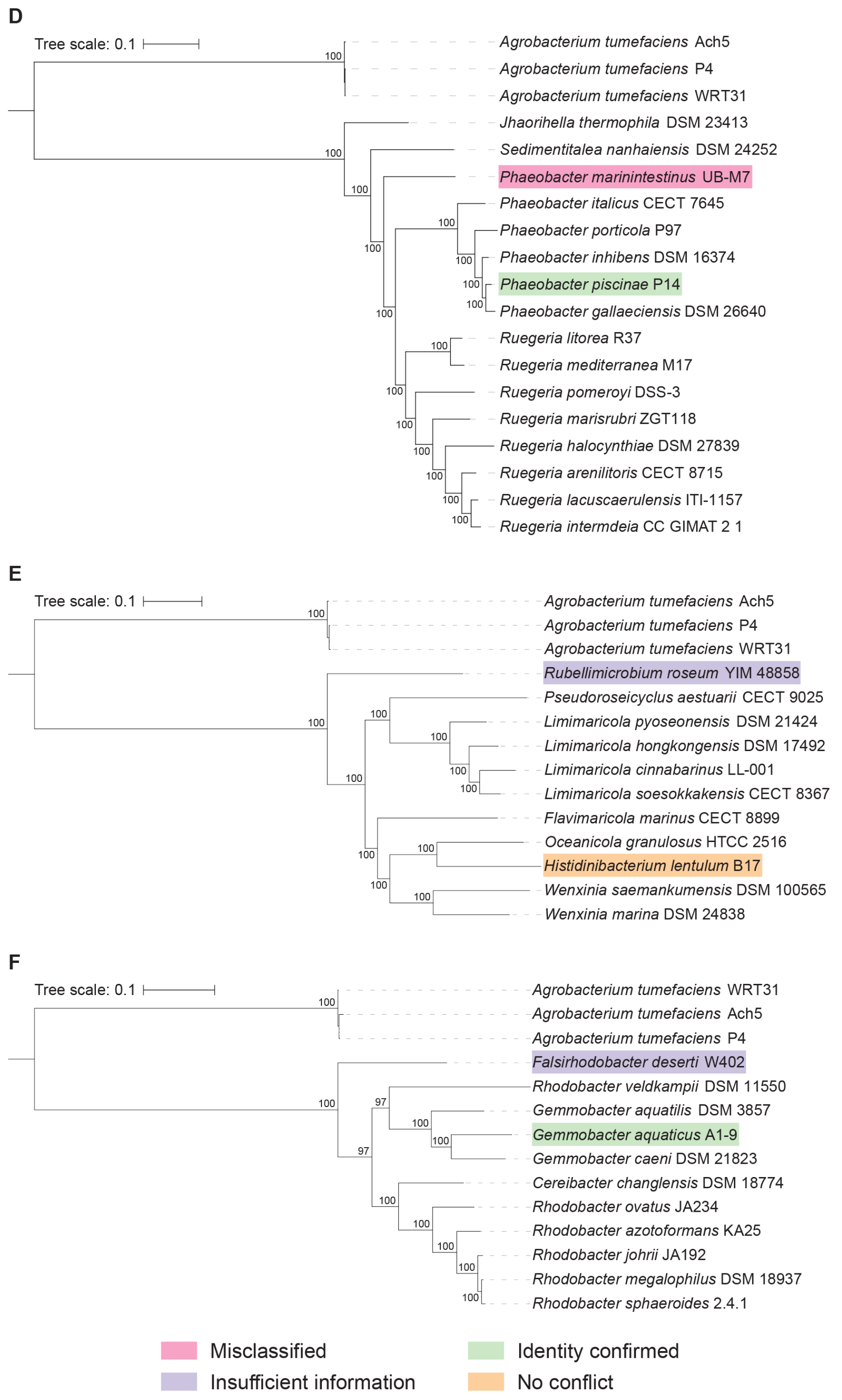

Supplementary Figure 7. (See legend on page 15)

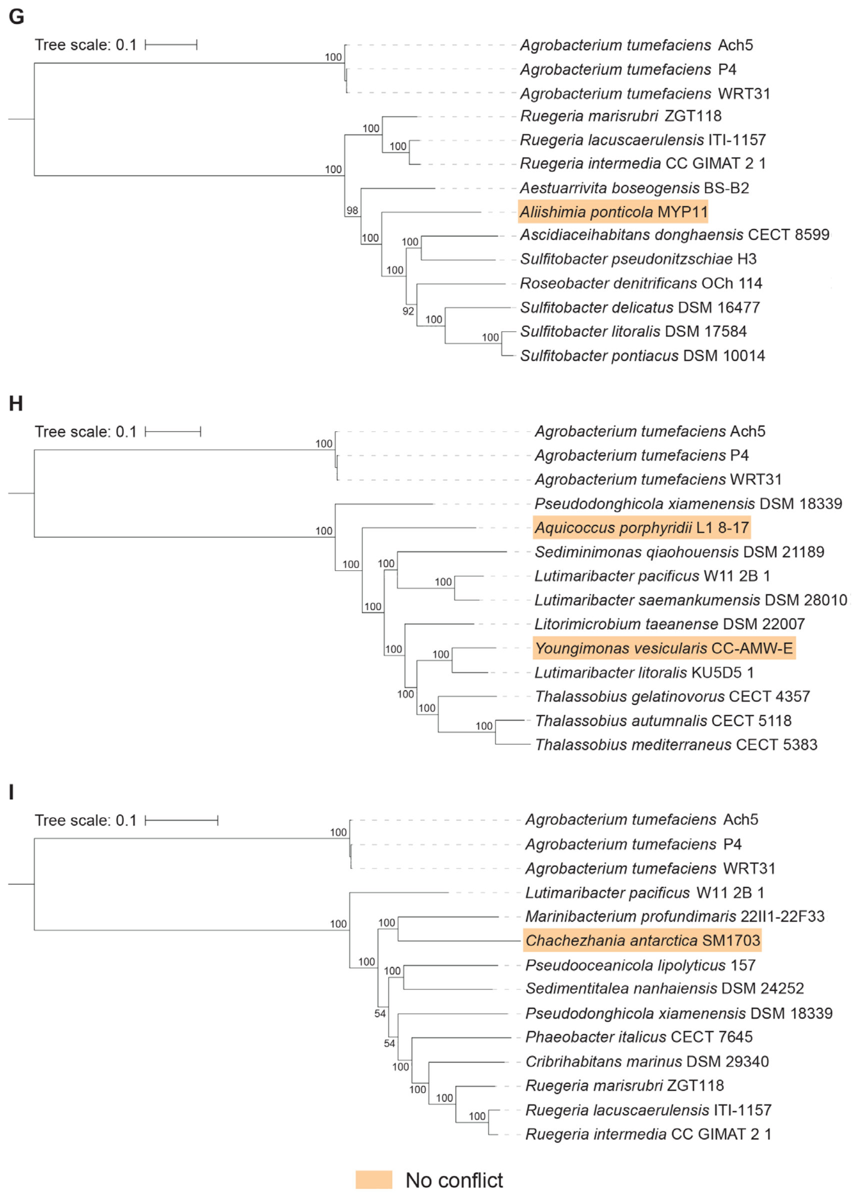

Supplementary Figure 7. (See legend on page 15)

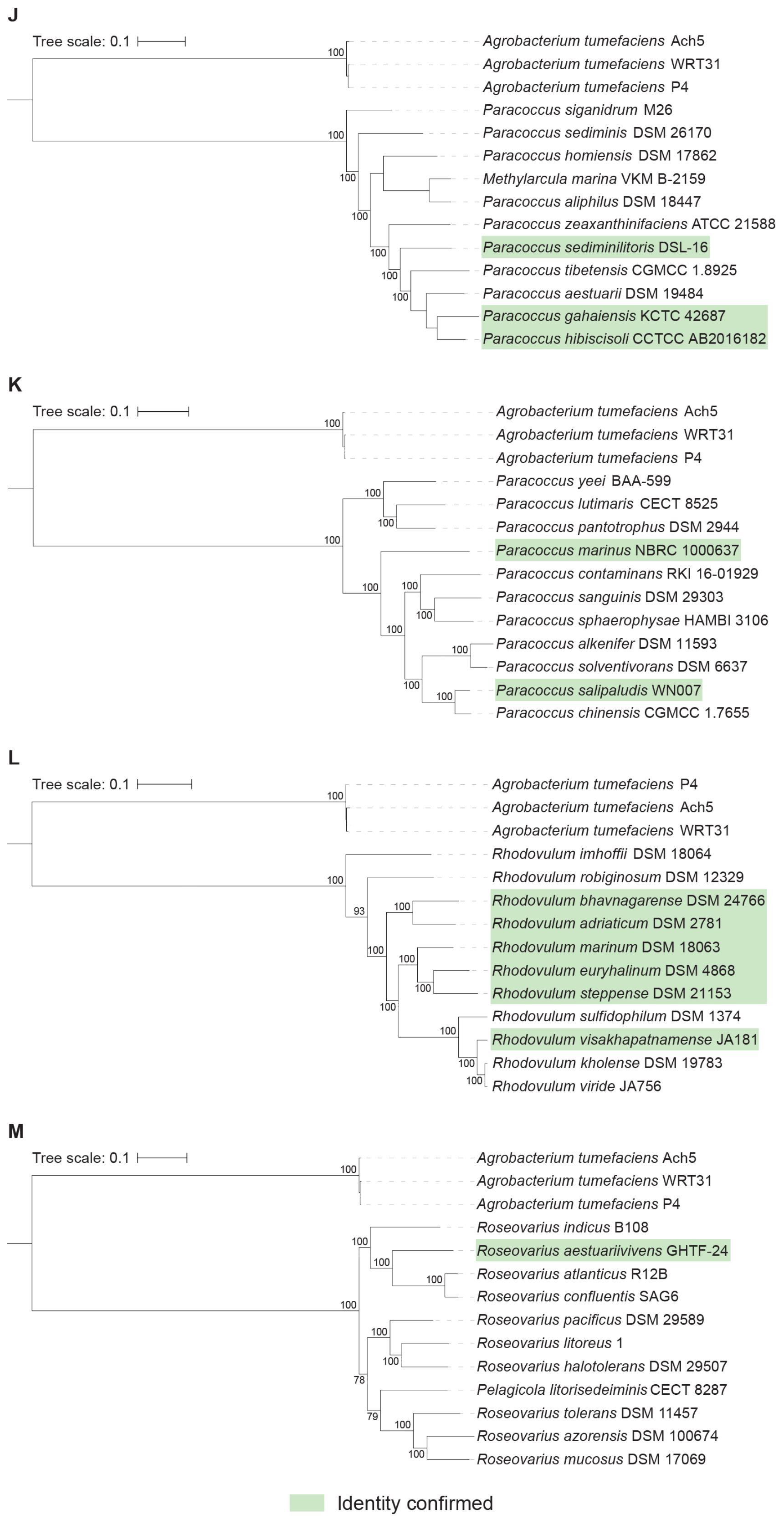

Supplementary Figure 7. (See legend on next page)

Supplementary Figure 7. The evaluation of the phylogenomically guided taxonomic workflow proposed in this study using newly available genomes. Phylogenetic trees show 29 additional genomes collected after the commencement of this study (i.e., genomes not included in the core genome tree in Figure 2). The strains are indicated as either confirmed (green), misclassified and needs reclassification (pink), no change as the strain is currently the sole species of its genus but is not causing any para- or polyphyly (orange), or no change as there is insufficient information for reclassification (purple). The maximum-likelihood trees were reconstructed using RAxML 8.2.11 with the PROTGAMMAAUTO option for automatic model selection and rooted with three *A. tumefaciens* strains. Branch support is evaluated with 100 bootstrap replicates and indicated on the nodes. The scale bars represent amino acid substitutions per site.
